# DDR1 contributes to kidney inflammation and fibrosis by promoting the phosphorylation of BCR and STAT3

**DOI:** 10.1172/jci.insight.150887

**Published:** 2022-02-08

**Authors:** Corina M. Borza, Gema Bolas, Fabian Bock, Xiuqi Zhang, Favour C. Akabogu, Ming-Zhi Zhang, Mark de Caestecker, Min Yang, Haichun Yang, Ethan Lee, Leslie Gewin, Agnes B. Fogo, W. Hayes McDonald, Roy Zent, Ambra Pozzi

**Affiliations:** 1 Department of Medicine, Division of Nephrology and Hypertension, and; 2 Department of Pathology, Microbiology and Immunology, Vanderbilt University School of Medicine, Nashville, Tennessee, USA.; 3 Department of Cell and Developmental Biology, Vanderbilt University, Nashville, Tennessee, USA.; 4 Division of Nephrology, Washington University School of Medicine, St. Louis, Missouri, USA.; 5 Proteomics Laboratory, Mass Spectrometry Research Center, Vanderbilt University, Nashville, Tennessee, USA.; 6 Department of Biochemistry, Vanderbilt University School of Medicine, Nashville, Tennessee, USA.; 7 Department of Veterans Affairs, Nashville, Nashville, Tennessee, USA.

**Keywords:** Cell Biology, Nephrology, Fibrosis

## Abstract

Discoidin domain receptor 1 (DDR1), a receptor tyrosine kinase activated by collagen, contributes to chronic kidney disease. However, its role in acute kidney injury and subsequent development of kidney fibrosis is not clear. Thus, we performed a model of severe ischemia/reperfusion-induced acute kidney injury that progressed to kidney fibrosis in WT and *Ddr1*-null mice. We showed that *Ddr1*-null mice had reduced acute tubular injury, inflammation, and tubulointerstitial fibrosis with overall decreased renal monocyte chemoattractant protein (MCP-1) levels and STAT3 activation. We identified breakpoint cluster region (BCR) protein as a phosphorylated target of DDR1 that controls MCP-1 production in renal proximal tubule epithelial cells. DDR1-induced BCR phosphorylation or BCR downregulation increased MCP-1 secretion, suggesting that BCR negatively regulates the levels of MCP-1. Mechanistically, phosphorylation or downregulation of BCR increased β-catenin activity and in turn MCP-1 production. Finally, we showed that DDR1-mediated STAT3 activation was required to stimulate the secretion of TGF-β. Thus, DDR1 contributes to acute and chronic kidney injury by regulating BCR and STAT3 phosphorylation and in turn the production of MCP-1 and TGF-β. These findings identify DDR1 an attractive therapeutic target for ameliorating both proinflammatory and profibrotic signaling in kidney disease.

## Graphical abstract

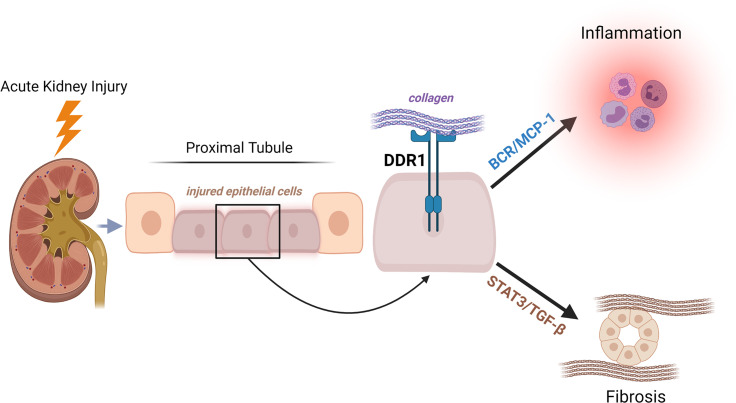

## Introduction

Severe acute kidney injury (AKI) has been linked to progression to chronic kidney disease (CKD) (1). Renal proximal tubule epithelial cells (RPTECs) are a target of acute tubular injury, which is the most common cause of AKI. RPTECs also contribute to the progression to CKD by producing proinflammatory cytokines, which promote inflammatory cell infiltration, and by stimulating myofibroblast differentiation of surrounding fibroblasts, thus leading to interstitial fibrosis (2). Multiple factors influence the secretion of proinflammatory and profibrotic cytokines by the injured RPTECs, including extracellular matrix receptors. Among these receptors, discoidin domain receptor 1 (DDR1) is a receptor tyrosine kinase that binds to and is activated by collagen. DDR1 is expressed at low levels in healthy organs; however, its expression and activation are increased in disease and after injury (3, 4). In CKD, DDR1 promotes inflammatory cell infiltration, secretion of profibrotic cytokines, and tissue fibrosis (5–9). Gene expression analysis of injured kidneys showed that DDR1 inhibitors revert the fibrotic and inflammatory gene networks activated by injury (3), pointing to DDR1 as a promising therapeutic target (4).

Collagen-mediated DDR1 activation initiates intracellular signaling pathways that affect fibrotic responses (10). DDR1 promotes fibrosis by increasing production of TGF-β (7) and by directly stimulating collagen transcription (11). We have previously showed that collagen-mediated DDR1 activation is a key step in promoting DDR1 interaction with nonmuscle myosin II (NM II) and β-actin and translocation to the nucleus. In the nucleus, DDR1 interacts with chromatin, thus favoring the transcription of collagen IV, a major collagen upregulated in fibrosis (11). DDR1 interaction with NM II is also required for the DDR1-mediated collagen fiber alignment and compaction, a process that contributes to tissue fibrosis (12). Although the profibrotic action of DDR1 has been investigated, how DDR1 promotes inflammatory cell infiltration and/or exerts a proinflammatory effect is less understood. In a unilateral ureter obstruction (UUO) model of kidney injury, it was proposed that DDR1 expressed on activated macrophages contributes to their migration to the site of injury (7). However, in angiotensin II–induced kidney injury, genetic deletion of DDR1 results in overall reduction of inflammatory cell infiltration, although DDR1 does not seem to be expressed on macrophages (9). Consistent with this study, DDR1 expression on macrophages was not detected in subjects with interstitial nephritis (4). These findings, together with the observation that DDR1 expression is detected in proximal tubules of patients with AKI who received a kidney transplant (11), suggest that DDR1 expressed on injured resident tubular epithelial cells, rather than on infiltrating macrophages, is responsible for regulating DDR1-dependent proinflammatory effects.

To better understand how DDR1 regulates proinflammatory and profibrotic signaling in kidney disease, we used a model of severe unilateral ischemia/reperfusion (IR) injury followed by delayed contralateral nephrectomy (13, 14). This approach improves postinjury survival and allows functional assessment of renal recovery after injury. In addition, it represents an excellent model to study inflammatory and fibrotic responses. We showed that *Ddr1*-null mice had significantly reduced AKI and inflammation and overall development of fibrosis. Mechanistically, activation of DDR1 on RPTECs led to phosphorylation of the breakpoint cluster region protein (BCR) and STAT3, 2 key steps in promoting the production of monocyte chemoattractant protein-1 (MCP-1) and TGF-β, respectively. Overall, we identified BCR and STAT3 as key players in DDR1-mediated proinflammatory and profibrotic effects after kidney injury.

## Results

### Loss of DDR1 reduces AKI and tubulointerstitial fibrosis in an AKI-to-CKD injury model.

**Figure 1:**
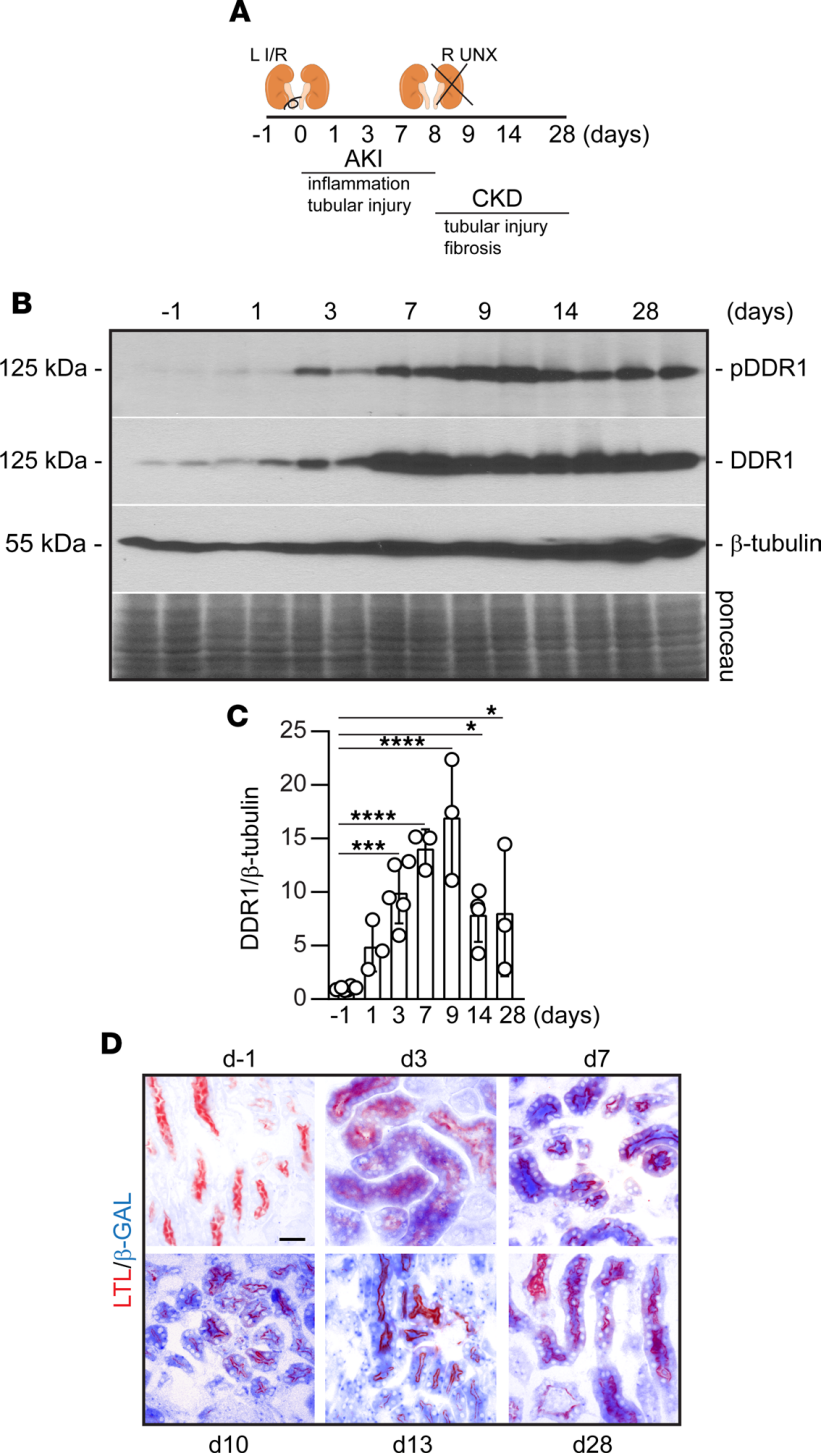
Increased DDR1 expression and activation after severe AKI that progresses to CKD. (**A**) Schematic representation of the injury model used in this study. Mice underwent left (L) renal pedicle clamping (IR) for 31 minutes, and nephrectomy of the right kidney (R UNX) was performed 8 days later. Mice were euthanized at the time points indicated. d–1 indicates preinjured mice. (**B**) Levels of phosphorylated and total DDR1 were analyzed by Western blot in kidney cortices isolated from uninjured (d–1) or injured WT mice at the time points indicated. (**C**) DDR1 and β-tubulin bands were quantified by densitometry, and values are expressed as DDR1/β-tubulin ratio and represent the mean ± SD of *n* ≥ 3 mice/group. Statistical analysis: 1-way ANOVA followed by Dunnett’s multiple-comparison test versus uninjured mice. *P < 0.05, ***P < 0.001, ****P < 0.0001. (**D**) Kidney sections from uninjured (d–1) or injured *Ddr1*^tm1a/tm1a^ mice (d–1 *n* = 3, d3 *n* = 4, d7 *n* = 3, d10 *n* = 1, d13 *n* = 1, d28 *n* = 3) euthanized at the time points indicated were stained for β-gal (blue staining) and lotus tetragonolobus lectin (LTL, a marker of proximal tubules, red staining) as described in the Methods. Note the increased expression of β-gal staining in injured proximal tubules. Scale bar: 25 μm.

To determine the contribution of DDR1 to AKI and development of fibrosis after AKI, we used an IR-induced AKI with delayed contralateral nephrectomy model. WT mice underwent left renal pedicle clamping for 31 minutes followed by nephrectomy of the right kidney 8 days later (Figure 1A). This method results in severe AKI in the first 7 days, which progresses to the development of tubulointerstitial fibrosis at d28 (13, 14). Analysis of renal DDR1 protein expression revealed that DDR1 levels and activation were evident at d3, peaked at d7–9, and were still upregulated at d28 (Figure 1, B and C). To visualize DDR1 localization and expression in uninjured and injured kidneys, we used *Ddr1*^tma1(EUCOMM)Hmgu^ mice that carry the *LacZ* gene between exons 5 and 6 of the *Ddr1* gene (Supplemental Figure 1A; supplemental material available online with this article; https://doi.org/10.1172/jci.insight.150887DS1). Because the *LacZ* gene is regulated by the endogenous *Ddr*1 promoter, β-gal staining can be used to localize DDR1 expression. For the injury, we generated *Ddr1*^tma1/tma1^ mice (Supplemental Figure 1B) that lacked endogenous expression of DDR1 (Supplemental Figure 1C). As shown in Figure 1D, low levels of β-gal activity were evident in the kidneys of uninjured mice (1 day prior to injury, d–1); however, positive staining was detected in proximal tubules starting at d3 that persisted for the entire duration of the injury (d28). Thus, DDR1 was upregulated in injured proximal tubule cells, the main cell type targeted by AKI. Moreover, the increased expression and activation of DDR1 during the acute and chronic phases of kidney injury suggest that DDR1 plays a role in both early and late stages of kidney injury.

**Figure 2:**
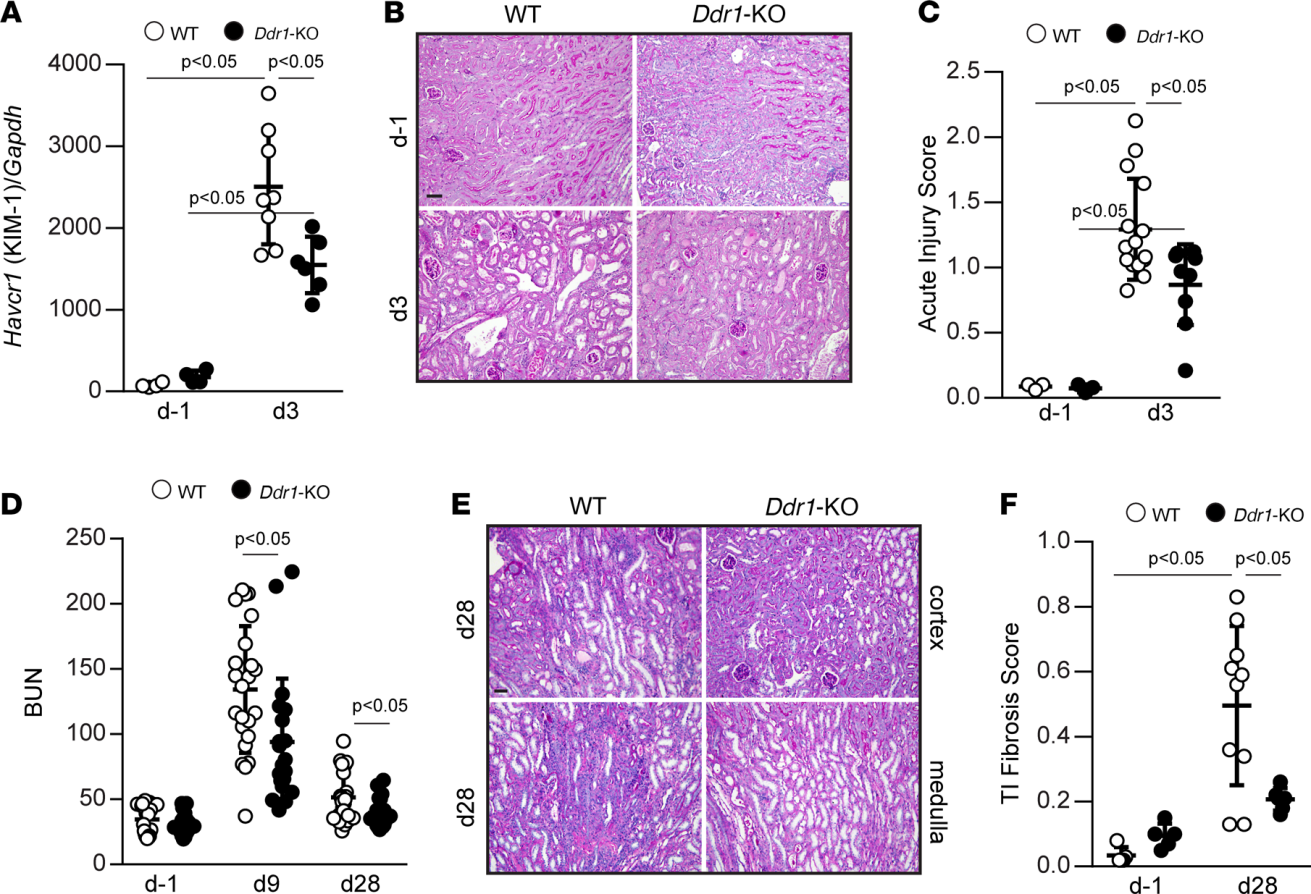
Attenuated AKI and chronic tubulointerstitial injury in *Ddr1-KO* mice. (**A**) *Havcr1* (KIM-1) mRNA levels in WT and *Ddr1-KO* mice uninjured (d–1) or 3 days after IR were analyzed by quantitative PCR and normalized to *Gapdh* mRNA. Circles represent individual kidneys, and the bars show mean ± SD. Uninjured WT and *Ddr1-KO* *n* = 4, injured WT *n* = 8, injured *Ddr1-KO* *n* = 6. (**B**) Periodic acid–Schiff (PAS) staining of kidneys from WT and *Ddr1-KO* mice uninjured (d–1) and d3 after IR. Scale bar: 50 μm. (**C**) Acute injury was evaluated in uninjured (d–1) (WT and *Ddr1-KO* *n* = 3) and d3 injured mice (WT *n* = 15, *Ddr1-KO* *n* = 9). Circles represent individual kidneys, and bars show mean ± SD. (**D**). Blood urea nitrogen (BUN) was measured prior to injury (d–1) and at d9 and d28 after injury. Circles represent individual mice, and bars show mean ± SD. (**E**) PAS staining of kidneys from WT and *Ddr1-KO* mice uninjured (d–1) and 28 days after injury. Scale bar: 50 μm. (**F**) Tubular injury scores were evaluated in uninjured (d–1) (WT and *Ddr1-KO* *n* = 5) and d28 injured (WT *n* = 10, *Ddr1-KO* *n* = 6) mice. Circles represent single mice, and bars are mean ± SD. Statistical analysis: 1-way ANOVA followed by Tukey’s multiple-comparison test for **A**, **C**, and **F** and 2-way repeated measures ANOVA followed by Sidak’s multiple-comparison test for **D**.

To determine how DDR1 participates in AKI, we examined kidney injury molecule-1 (KIM-1) at d3 after IR in injured WT and *Ddr1*-null (*Ddr1-KO*) mice. Renal *Havcr1* (KIM-1) mRNA levels increased at d3 in WT and *Ddr1-KO* mice, although they were significantly lower in the *Ddr1-KO* mice (Figure 2A). Consistent with this result, attenuated tubular injury, tubular necrosis, dilatation, and casts were evident in d3 injured *Ddr1-KO* mice compared with WT mice (Figure 2, B and C).

**Figure 3:**
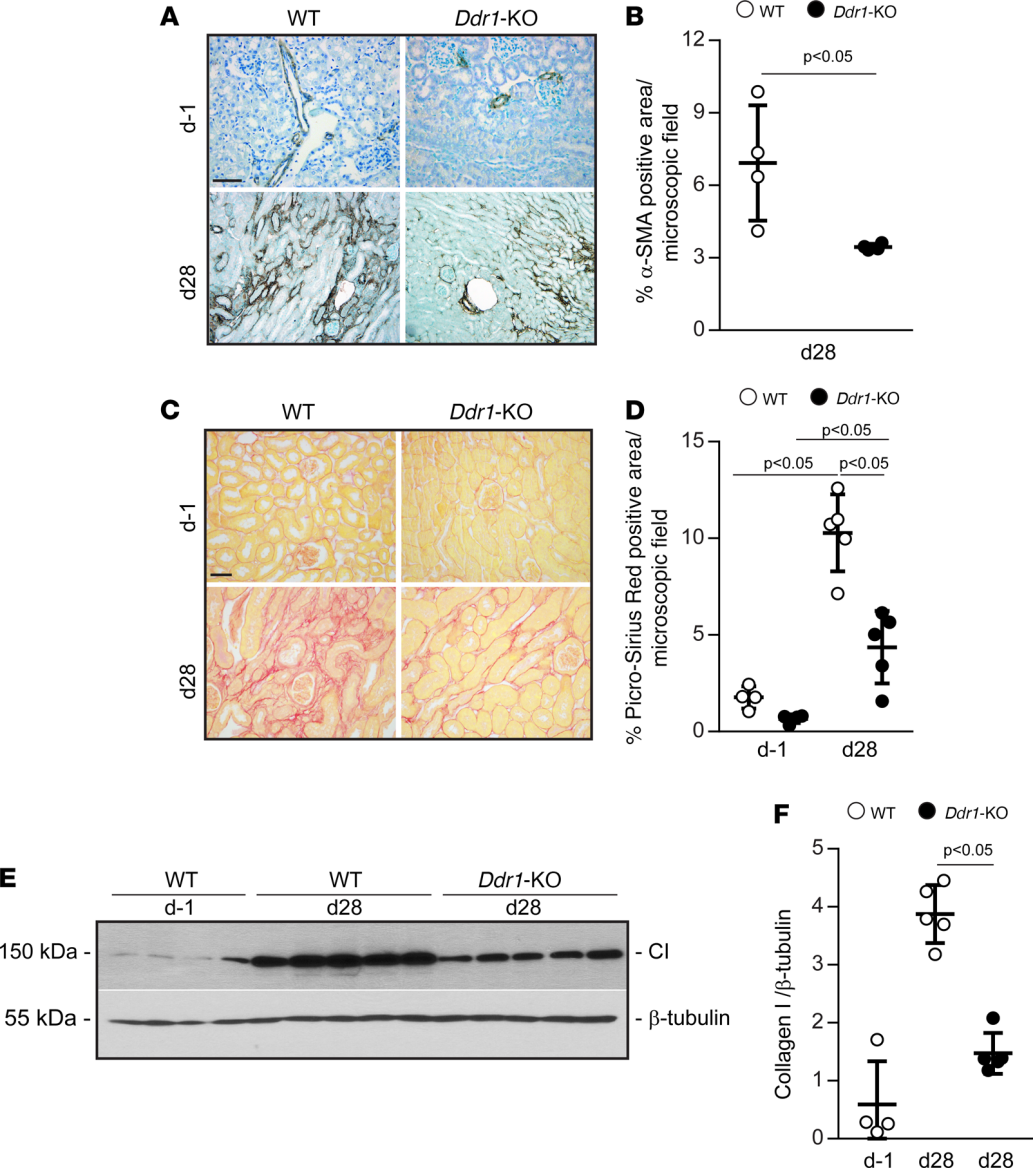
Decreased fibrosis in injured *Ddr1-KO* mice. (**A**) Images of kidney sections from WT and *Ddr1-KO* mice uninjured (d–1) or 28 days after IR stained with anti–α-SMA antibody. Scale bar: 50 μm. (**B**) The percentage of α-SMA–positive area per microscopic field was evaluated at d28 using ImageJ. Circles represent individual kidneys (WT and *Ddr1-KO* *n* = 4), and bars are mean ± SD. (**C**) Picrosirius red staining of kidney sections from WT and *Ddr1-KO* mice uninjured (d–1) or 28 days after IRI. Scale bar: 50 μm. (**D**) The percentage of Picrosirius red–positive area was evaluated using ImageJ. Circles represent individual kidneys (d–1 WT and *Ddr1-KO* *n* = 4, d28 WT and *Ddr1-KO* *n* = 5), and bars are mean ± SD. (**E**) Kidney lysates from uninjured WT (d–1) and WT and *Ddr1-KO* 28 days after IR were analyzed for the level of collagen I by Western blot analysis. (**F**) Collagen I and β-tubulin bands were quantified by densitometry analysis, and collagen I is expressed as collagen I/β-tubulin ratio. Circles represent individual kidneys (d–1 WT *n* = 4, d28 WT and *Ddr1-KO* *n* = 5), and bars are mean ± SD. Statistical analysis: 2-tailed *t* test for **B** and 1-way ANOVA followed by Tukey’s multiple-comparison test for **D** and **F**.

To assess how DDR1 influences the development of fibrosis after AKI, we measured serum blood urea nitrogen (BUN) in WT and *Ddr1-KO* mice at d–1, at d9 (immediately after removal of the contralateral kidney), and at d28 (Figure 2D). Both WT and *Ddr1-KO* mice showed increased BUN at d9 compared with d–1, although it was significantly lower in the injured *Ddr1-KO* mice. At d28, BUN in the *Ddr1-KO* mice returned to baseline levels, whereas it remained significantly higher in the injured WT mice (Figure 2D). Consistent with this result, kidneys of WT mice showed increased tubulointerstitial fibrosis, tubular atrophy, and interstitial cell infiltration compared with those of *Ddr1-KO* mice at d28 (Figure 2, E and F). Further, WT kidneys at d28 showed significantly higher levels of cells positive for α–smooth muscle actin (α-SMA; indicative of myofibroblast differentiation) (Figure 3, A and B) and collagen deposition as determined by Picrosirius red staining (Figure 3, C and D) and by Western blot analysis (Figure 3, E and F) compared with injured *Ddr1-KO* mice. These results suggest that DDR1 contributes to AKI and development of fibrosis.

### Loss of DDR1 reduced macrophage infiltration and MCP-1 production after IR.

**Figure 4:**
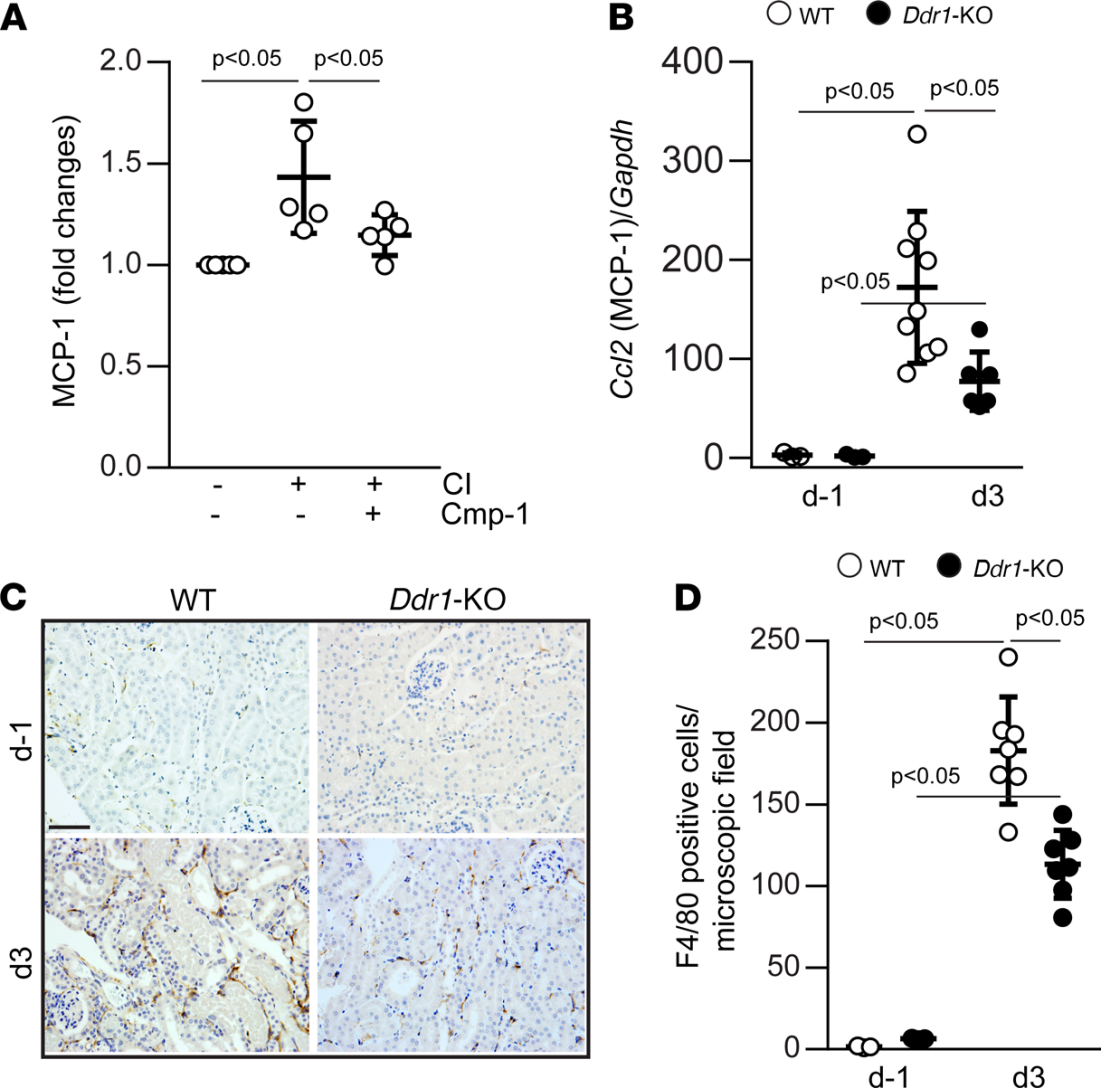
DDR1 promotes the production of proinflammatory MCP-1. (**A**) MCP-1 levels were measured by ELISA in conditioned medium of RPTECs treated with vehicle or collagen I (CI, 50 μg/mL) with or without the DDR1 inhibitor Cmp-1 (3 μM). Each circle represents 1 experiment performed in triplicates. Data represent mean ± SD of 5 experiments and are expressed as fold-change relative to vehicle-treated cells assigned a value of 1. (**B**) *Ccl2* (MCP-1) mRNA levels were measured in kidneys of uninjured (d–1) or 3 days injured WT and *Ddr1-KO* mice by real-time quantitative PCR and normalized to *Gapdh* mRNA. Circles represent individual kidneys (d–1 WT and *Ddr1-KO* *n* = 3, d3 WT *n* = 9, d3 *Ddr1-KO* *n* = 6), and bars are mean ± SD. I Images of kidney sections from uninjured (d–1) or 3 days injured WT and *Ddr1-KO* mice stained with anti-F4/80 antibody. Scale bar: 50 μm. (**D**) The number of F4/80 positive cells per microscopic field was evaluated and expressed as F4/80-positive cells/microscopic field. Circles represent individual kidneys (d–1 WT and *Ddr1-KO* *n* = 3, d3 WT and *Ddr1-KO* *n* = 7), and bars are mean ± SD. Statistical analysis: 1-way ANOVA followed by Dunnett’s multiple comparison versus CI-treated cells for **A** and 1-way ANOVA followed by Tukey’s multiple-comparison test for **B** and **D**.

Proximal tubules, the main target of acute tubular injury in this model, are critical contributors to the development of CKD (15), which is due in part to synthesis of proinflammatory cytokines. DDR1 is expressed in proximal tubules of patients with AKI who received a transplant (11) as well as mouse RPTECs (Supplemental Figure 2, A and B). In RPTECs, endogenous DDR1 is phosphorylated in response to collagen stimulation, and this effect is inhibited by the small-molecule DDR1 inhibitor Cmp-1 (16, 17) (Supplemental Figure 2, A and B). To determine whether activated DDR1 in RPTECs regulates the secretion of proinflammatory cytokines, we analyzed the conditioned medium of vehicle-treated versus collagen-treated RPTECs using a proinflammatory cytokine array. Several cytokines were upregulated in response to collagen treatment. MCP-1 showed the strongest signal and most upregulation in response to collagen (Supplemental Figure 2C). These results were confirmed by ELISA showing significant upregulation of MCP-1 levels in collagen-treated versus vehicle-treated cells (Figure 4A), and this induction was significantly decreased in cells treated with Cmp-1 (Figure 4A). These findings suggest that activation of DDR1 promotes MCP-1 production in RPTECs. To confirm the in vitro results, we measured *Ccl2* (MCP-1) mRNA in the kidneys of WT and *Ddr1-KO* mice 3 days after severe IR. MCP-1 levels were upregulated in injured WT mice; there was significantly less increase in the injured *Ddr1-KO* mice (Figure 4B). Infiltrating F4/80-positive cells were significantly decreased in d3 injured *Ddr1-KO* mice compared with WT mice, consistent with decreased renal MCP-1 levels (Figure 4, C and D).

### Identification of BCR as a DDR1 target using a proximity-dependent biotinylation assay.

To determine how DDR1 contributes to MCP-1 production, we performed a proximity-dependent biotinylation assay, BioID, to identify possible DDR1 interacting partners that regulate inflammatory responses. BioID relies on the biotinylation of potential interactors in live cells by a promiscuous bacterial protein ligase fused to the bait protein. The proteins thus identified represent direct and indirect interactors as well as proteins in close proximity to the bait. To do this, we generated HEK cells expressing DDR1 linked to 2 forms of the biotin ligase, namely BirA* (DDR1-BioID) or turbo-Flag (DDR1-turbo-Flag) (Supplemental Figure 3A). We verified that the DDR1-BioID was phosphorylated in response to collagen (Supplemental Figure 3B) and that the biotin ligase was active (Supplemental Figure 3C). Biotinylated proteins from cells expressing empty vector (BioID) treated with collagen I for 24 hours or from cells expressing the biotin ligase fused to DDR1 (DDR1-BioID), treated with vehicle or collagen for 24 hours, were isolated on neutravidin beads and subjected to mass spectrometry analysis (see Methods for details). Several proteins were detected in cells expressing DDR1-BioID but not in BioID alone (Supplemental Table 1). As expected, DDR1 was the most abundant protein detected in vehicle-treated and collagen-treated DDR1-BioID cells (Supplemental Table 1). Several proteins were biotinylated in the collagen-treated versus vehicle-treated DDR1-BioID cells, with BCR significantly different between the 2 treatment groups.

**Figure 5:**
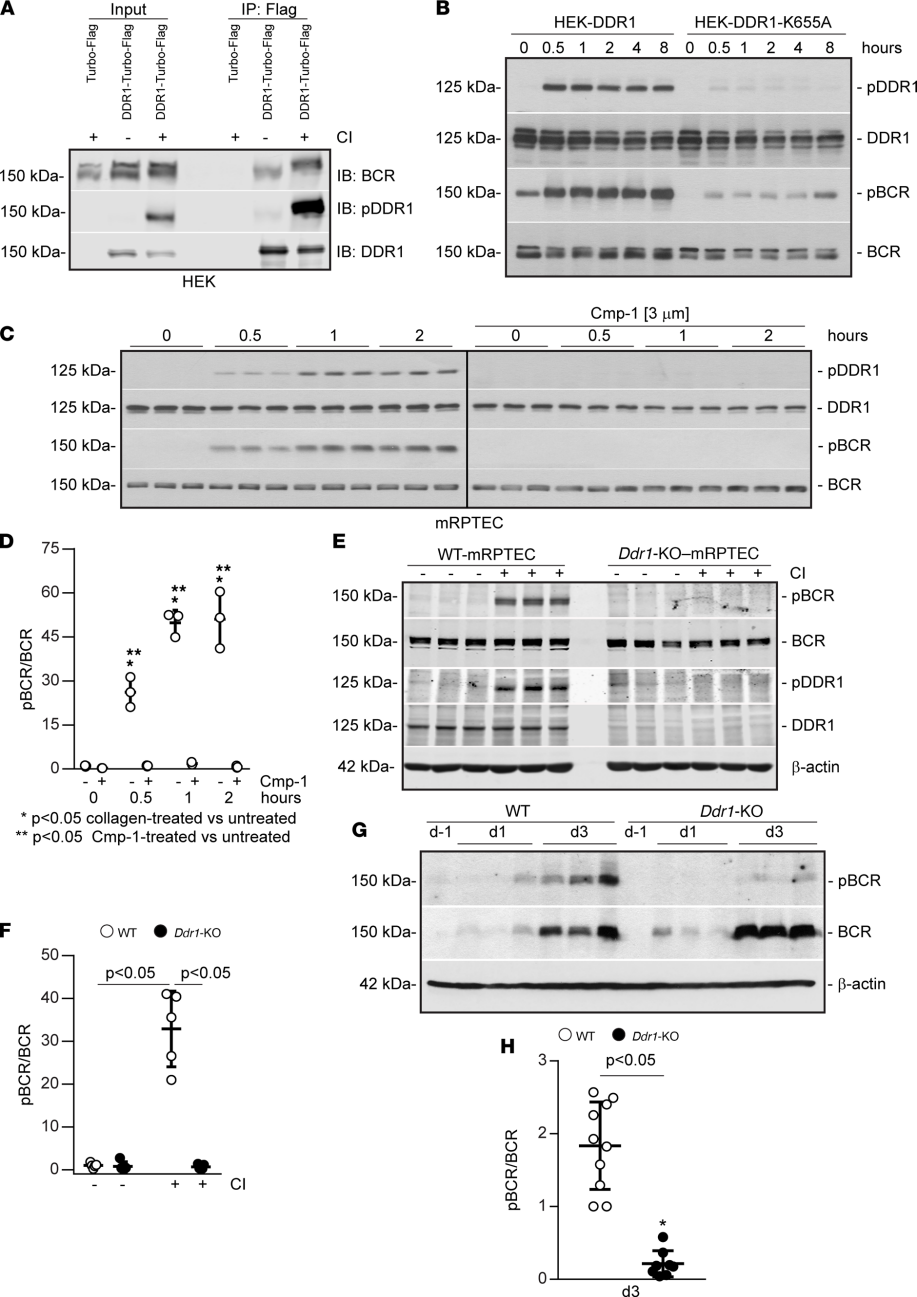
DDR1 promotes BCR phosphorylation in vivo and in vitro. (**A**) Vector- or DDR1-HEK cells were treated with collagen I (50 μg/mL) or vehicle, then lysed and immunoprecipitated with anti-Flag antibody, and analyzed for levels of total and phosphorylated DDR1 or BCR. (**B**) DDR1-HEK or DDR1-K655A-HEK cells were treated with collagen I (50 μg/mL), and the cell lysates were analyzed for levels of total and phosphorylated DDR1 or BCR. (**C**) RPTECs were treated with collagen I (50 μg/mL) ± Cmp-1 as indicated and then analyzed as described in **B**. The black vertical line separates 2 gels that were run and developed at the same time. (**D**) pBCR and BCR bands in **C** were quantified by densitometry, and the pBCR level is expressed as pBCR/BCR ratio. Dara shown are mean ± SD of 1 experiment performed in triplicate (*n* = 3 experiments). Statistical analysis: 1-way ANOVA followed by Dunnett’s multiple-comparison test versus untreated cells for pBCR and 2-way ANOVA followed by Sidak’s multiple-comparison test for Cmp-1–treated versus untreated cells. (**E**) Primary RPTECs isolated from WT (WT-mRPTECs) and *Ddr1-KO* (*Ddr1-KO*-mRPTECs) mice were treated with vehicle or collagen I (50 μg/mL) for 30 minutes and then analyzed as described above. (**F**) pBCR and BCR bands were quantified using the software provided by Odyssey CLx imaging system. Circles represent cells isolated from a single mouse and values represent mean ± SD *n* = 5 mice for each group. Statistical analysis: 1-way ANOVA followed by Tukey’s multiple-comparison test. (**G**) Kidney cortices from uninjured (d–1), d1, and d3 injured WT and *Ddr1-KO* mice were analyzed by Western blot for the levels of pBCR and BCR. (**H**) pBCR and BCR bands at d3 were quantified by densitometry, and pBCR is expressed as pBCR/BCR ratio. Circles represent individual kidneys, values represent mean ± SD, WT *n* = 10, *Ddr1-KO* *n* = 8. Statistical analysis: 2-tailed *t* test.

To confirm the mass spectrometry results, we isolated biotinylated proteins from BioID cells treated with collagen I or from DDR1-BioID cells treated with vehicle or collagen I and performed Western blot analysis with an anti-BCR antibody. BCR was not detected in BioID-cells (BioID) and present at low levels in vehicle-treated DDR1-BioID cells. In contrast, biotinylated BCR was robustly detected in collagen-treated DDR1-BioID cells (Supplemental Figure 3D). This result confirms that BCR was in close proximity to DDR1 after collagen activation. In addition, IP assays in vehicle- and collagen-treated HEK cells expressing DDR1-Turbo-Flag with anti-FLAG antibody showed that BCR was coimmunoprecipitated with DDR1 in vehicle-treated and collagen-treated cells, although the amount of immunoprecipitated BCR was more predominant in collagen-treated cells (Figure 5A).

### Reduced levels of phosphorylated BCR in Ddr1-KO mice after IR.

Quantitative phosphoproteomics identified BCR as a protein phosphorylated on Tyr177 in response to DDR1 activation (18). Tyr177 phosphorylation is a key step for regulation of BCR function (19–21). To determine whether collagen-induced DDR1 activation regulates BCR phosphorylation, we examined the levels of pTyr177BCR in HEK cells expressing DDR1 (HEK-DDR1). BCR phosphorylation was detected 30 minutes after collagen treatment, and it remained elevated up to 8 hours and paralleled collagen-mediated DDR1 autophosphorylation (Figure 5B). Furthermore, DDR1-induced BCR phosphorylation was greatly reduced in HEK cells expressing the kinase-deficient DDR1 (HEK-DDR1-K655A) (Figure 5B). We next confirmed that DDR1 induces BCR phosphorylation in RPTECs also, as it was phosphorylated upon collagen treatment and prevented by treatment with Cmp-1 (Figure 5, C and D). To confirm the inhibitor results, we isolated primary RPTECs from WT (WT-mRPTEC) and *Ddr1-KO* (*Ddr1-KO*-mRPTEC) mice using a collagenase/Dispase digestion method (See Methods and Supplemental Figure 4A). Both WT and *Ddr1-KO* mRPTECs express typical markers of proximal tubule cells, including epithelial ZO-1, aquaporin-1, N-cadherin, and Claudin-2 (Supplemental Figure 4, B and C). Importantly, WT-mRPTECs express both DDR1 and BCR, whose phosphorylation can be induced by collagen treatment and inhibited by Cmp-1 (Supplemental Figure 4, D–F). Consistent with these results, analysis of WT-mRPTECs and *Ddr1-KO*-mRPTECs showed that BCR was phosphorylated only in WT but not *Ddr1-KO* cells after treatment with collagen (Figure 5, E and F). All together, these results indicate that BCR is a downstream target of collagen-activated DDR1 in proximal tubule cells.

Next, we determined whether BCR is phosphorylated in our AKI model. To do this, we examined WT and *Ddr1-KO* kidney tissue lysates at d–1, d1, and d3 after severe IR. BCR protein levels were increased in d3 injured WT and *Ddr1-KO* mice, although phosphorylated BCR was primarily detected in d3 injured WT mice (Figure 5, G and H). Interestingly, d3 after IR was also the time point of significant increase in DDR1 levels and activation (Figure 1B), suggesting that BCR might function downstream of activated DDR1.

### BCR inhibits MCP-1 production by regulating β-catenin activation.

**Figure 6:**
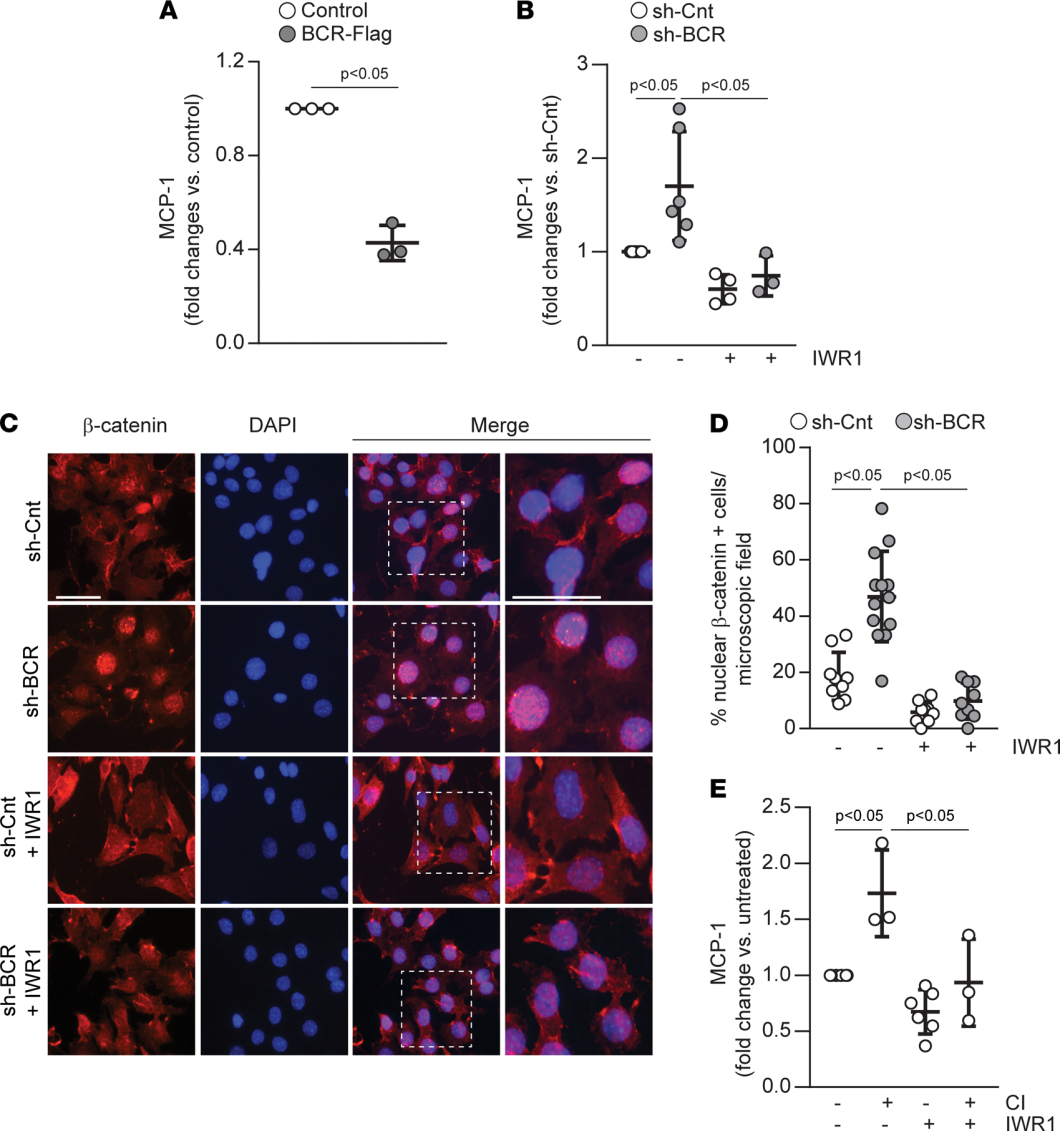
BCR negatively regulates MCP-1 production by inhibiting β-catenin activity. (**A**) MCP-1 levels were measured by ELISA in 24-hour conditioned medium of RPTECs overexpressing BCR (BCR-Flag). Each circle represents an independent experiment performed in triplicate, and values represent mean ± SD of 3 experiments and represent fold-change relative to control cells assigned a value of 1. Statistical analysis: 2-tailed *t* test. (**B**) MCP-1 levels were evaluated by ELISA in 24-hour conditioned medium of sh-Cnt or sh-BCR RPTECs treated with vehicle or collagen I (50 μg/mL) in the presence or absence of the β-catenin inhibitor IWR1-endo (30 μM). Each circle represents an independent experiment performed in triplicate. Values are mean ± SD of at least 3 experiments and represent fold-changes relative to vehicle-treated control assigned a value of 1. Statistical analysis: 1-way ANOVA followed by Tukey’s multiple-comparison test. (**C**) Representative images of sh-Cnt or sh-BCR RPTECs incubated with or without the β-catenin inhibitor IWR-1-endo (30 μM) and stained with anti–β-catenin antibody. Scale bar: 15 μm. (**D**) Percentage of nuclear β-catenin–positive cells. Circles represent values of single images, and bars show mean ± SD with a minimum of 400 cells counted in 2 independent experiments. (**E**) MCP-1 levels were measured by ELISA in 24-hour conditioned medium of RPTECs treated with vehicle or collagen I (CI, 50 μg/mL) in the presence or absence of IWR1-endo (30 μM). Each circle represents an independent experiment performed in triplicate. Values are mean ± SD of at least 3 experiments and represent fold-change relative to vehicle-treated cells assigned a value of 1. Statistical analysis: 2-tailed *t* test for **A** and 1-way ANOVA followed by Tukey’s multiple-comparison test for **B**, **D**, and **E**.

Because BCR has been involved in inflammation (22), we investigated whether the DDR1/BCR axis plays a role in MCP-1 production. Thus, we generated RPTECs either overexpressing (BCR-Flag) or downregulated for (sh-BCR) BCR (Supplemental Figure 5, A and B) and measured secreted MCP-1 levels in these cells at baseline. Surprisingly, we found that increasing BCR expression in RPTECs decreased the levels of secreted MCP-1 (Figure 6A), suggesting that BCR is a negative regulator of MCP-1 production. Consistent with this hypothesis, we found that decreasing the levels of BCR in sh-BCR RPTECs increased MCP-1 levels (Figure 6B).

We next determined how BCR negatively regulates MCP1 production. Because BCR activation increases NF-κB transcriptional activity (22, 23), we examined whether DDR1 controls nuclear translocation of NF-κB. To do this, we prepared nuclear fractions from vehicle-treated and collagen-treated HEK-DDR1 cells or RPTECs and examined the levels of NF-κB-p65. Nuclear NF-κB was not different in vehicle-treated versus collagen-treated HEK-DDR1 cells or RPTECs (Supplemental Figure 6, A and B) despite increased collagen-mediated BCR phosphorylation (Supplemental Figure 6A). In contrast, nuclear NF-κB was increased in cells treated with TNF-α or PMA, known to induce nuclear translocation of this transcription factor (Supplemental Figure 6, A and B). Consistent with this finding, nuclear levels of NF-κB were not different in the kidneys of d3 injured WT versus *Ddr1-KO* mice (Supplemental Figure 6, C and D). These results suggest that an alternative mechanism is responsible for BCR-mediated regulation of MCP-1 production in RPTECs.

BCR regulates WNT signaling by forming a complex with β-catenin and inhibiting β-catenin–dependent transcription (19). Phosphorylation of BCR at Tyr177 blocks the interaction with β-catenin and reverses BCR-dependent suppressive effects (19). To determine whether BCR levels affect nuclear β-catenin levels (indicative of WNT pathway activation), we analyzed the localization of β-catenin in RPTECs expressing control (sh-Cnt) or BCR shRNA (sh-BCR) cultured with or without the WNT/β-catenin inhibitor IWR-1-endo, which promotes β-catenin degradation by stabilizing the destruction complex component, Axin (24, 25). Nuclear β-catenin–positive cells were significantly increased in sh-BCR-RPTECs versus sh-Cnt-RPTECs (Figure 6, C and D). Treatment with IWR-1-endo significantly decreased the numbers of β-catenin–positive cells in both groups (Figure 6, C and D); the most effect was observed in the sh-BCR-RPTECs. Next, we addressed whether the increase in MCP-1 production in sh-BCR-RPTECs was due to the alleviation of the inhibitory effects of BCR on β-catenin. Treatment of sh-BCR-cells with IWR-1-endo significantly decreased the level of secreted MCP-1 to that of untreated sh-Cnt-cells (Figure 6B), suggesting that increased β-catenin activity is responsible for increased MCP-1 production in sh-BCR-RPTECs. Finally, to determine whether collagen-stimulated MCP-1 secretion is due to DDR1-mediated β-catenin activation, we measured secreted MCP-1 levels in collagen-treated RPTECs cultured with or without IWR1-endo. Inhibition of β-catenin activity significantly inhibited MCP-1 secretion in collagen-stimulated cells, suggesting that β-catenin is a key mediator of DDR1-induced MCP-1 production (Figure 6E). Thus, collagen-induced DDR1 activation results in BCR phosphorylation, which in turn leads to β-catenin–mediated MCP-1 production.

### DDR1 activation increases TGF-β production by activating STAT3.

**Figure 7:**
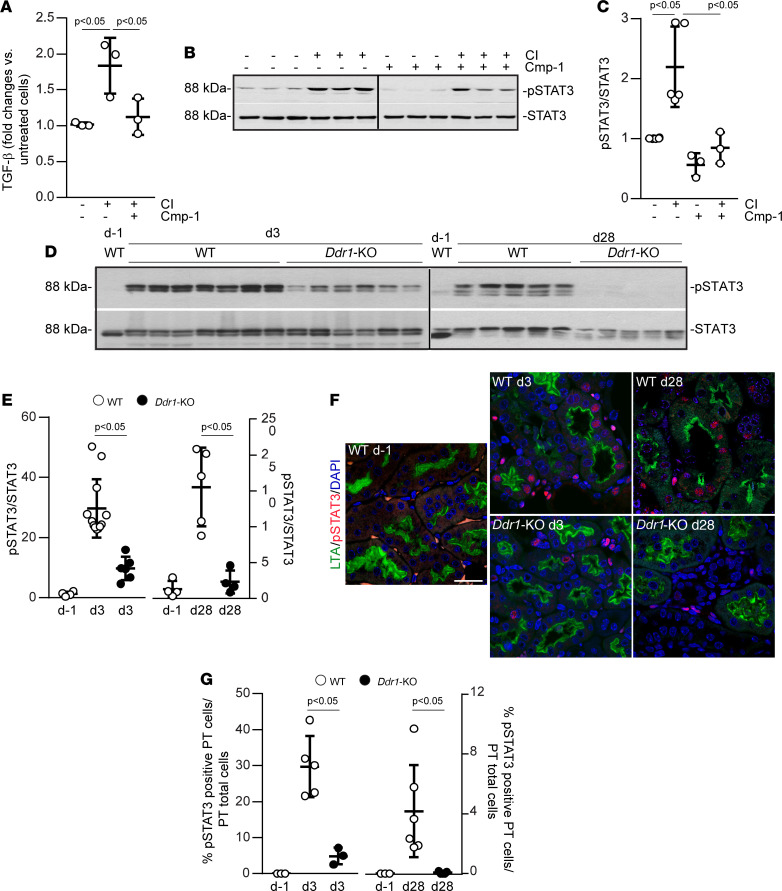
DDR1 activation promotes TGF-β production and STAT3 phosphorylation. (**A**) TGF-β was measured by ELISA in conditioned medium of RPTECs treated ± collagen I (CI) ± Cmp-1 (3 μM). Circles represent a single experiment performed in triplicate. Values are mean ± SD of 3 experiments and represent fold-changes versus vehicle-treated cells assigned as 1. Statistical analysis: 1-way ANOVA followed by Dunnett’s multiple-comparison test versus CI-treated group. (**B**) RPTECs were treated ± CI ± Cmp-1 (3 μM) and then analyzed by Western blot for phosphorylated (Tyr705) and total STAT3. The black vertical line separates 2 gels that were run and developed at the same time. (**C**) pSTAT3 and STAT3 bands were quantified by densitometry and pSTAT3 is expressed as pSTAT3/STAT3 ratio. Circles and values are as in **A**. Statistical analysis: 1-way ANOVA followed by Tukey’s multiple-comparison test. (**D** and **E**) Kidney cortices from uninjured (d–1), d3, and d28 injured WT and *Ddr1-KO* mice were analyzed by Western blot for levels of pSTAT3 and STAT3. The black vertical line separates 2 different gels. pSTAT3/STAT3 ratio was calculated as described in **C**. Circles represent an individual kidney (d–1 WT *n* = 4, d3 WT and *Ddr1-KO* *n* = 12, d28 WT and *Ddr1-KO* *n* = 5). Values are mean ± SD and represent fold-change versus d–1 assigned as 1. Statistical analysis was performed as in **C**. (**F** and **G**) Kidney sections from uninjured (d–1), d3, and d28 injured WT and *Ddr1-KO* mice were stained with anti-pSTAT3 antibody and LTL. Scale bar: 20 μm. The number of pSTAT3-positive and total number of proximal tubule cells was evaluated and expressed as described in the Methods. Circles represent a single kidney. Values represent mean ± SD (d–1 WT *n* = 3, d3 WT *n* = 5, d3 *Ddr1-KO* *n* = 3, d28 WT *n* = 6, d28 *Ddr1-KO* *n* = 5). Statistical analysis was performed as in **C**.

In addition to inflammatory cytokines, injured RPTECs secrete profibrotic cytokines like TGF-β. To determine whether DDR1 activation contributes to TGF-β secretion, we measured the levels of secreted TGF-β in vehicle-treated versus collagen-treated RPTECs using ELISA (Figure 7A). Collagen treatment significantly increased TGF-β production compared with vehicle-treated cells, which was prevented by treating the cells with the DDR1 inhibitor Cmp-1 (Figure 7A).

To investigate how DDR1 signaling promotes TGF-β production, we screened for kinases that were phosphorylated in response to collagen-induced DDR1 activation using a kinase array. For this assay, we used HEK-vector or HEK-DDR1 cells treated with vehicle or collagen. Transcription factor STAT3 phosphorylated on Tyr705 and on Ser727 was notable among the kinases phosphorylated only in collagen-treated HEK-DDR1 cells (Supplemental Figure 7, A and B). We confirmed DDR1-mediated STAT3 phosphorylation on Tyr705 in RPTECs treated with collagen in the presence or absence of the DDR1 inhibitor Cmp-1 (Figure 7, B and C). In addition, STAT3 was phosphorylated on Tyr705 in kidneys after IR-mediated injury, and it was more phosphorylated in 3d and 28d injured WT as compared with *Ddr1-KO* mice (Figure 7, D and E). IHC performed on uninjured and injured kidneys revealed increased pSTAT3 staining, primarily in d3 injured proximal tubules of WT but not *Ddr1-KO* mice (Figure 7, F and G). At d28, significantly more activated STAT3 was still observed in the kidneys of WT mice compared with *Ddr1-KO* mice (Figure 7, F and G); however, positive staining was observed in both proximal tubular and nontubular compartments (Figure 7F).

**Figure 8:**
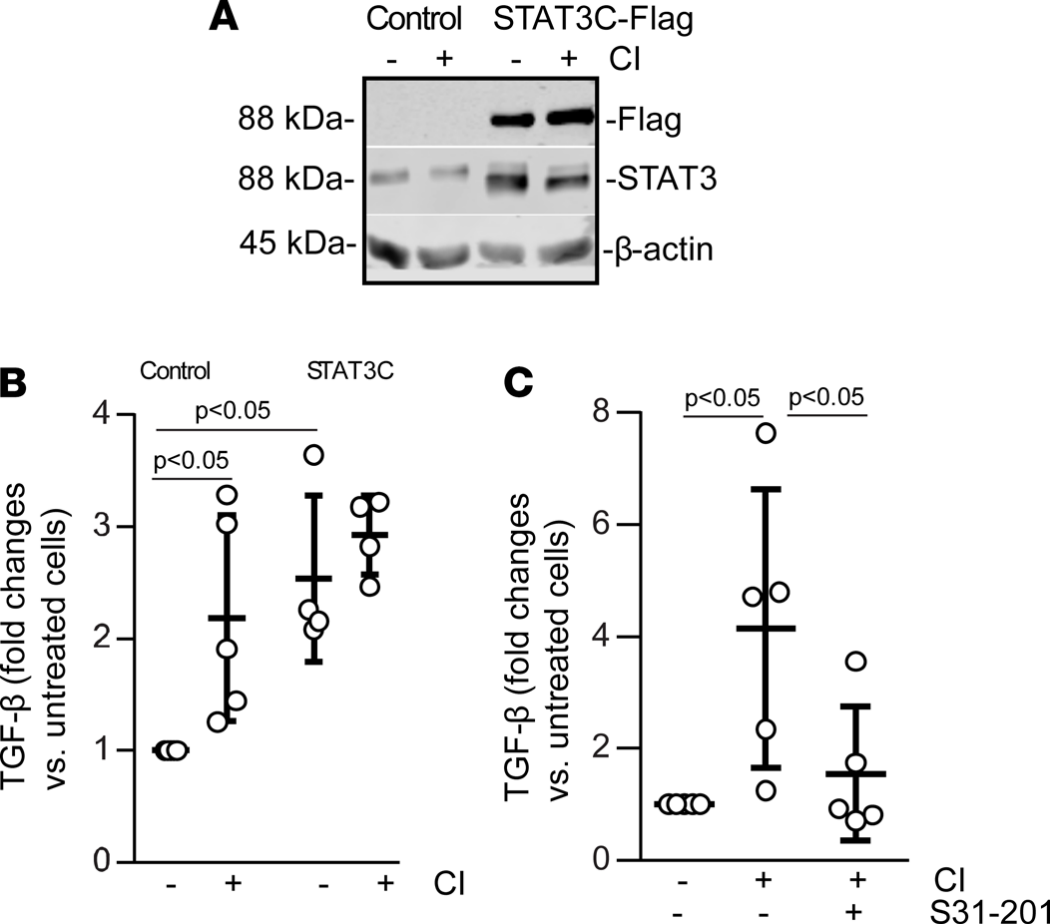
DDR1 activation promotes TGF-β production by activating STAT3. (**A**) Cell lysates of RPTECs expressing or not STAT3C-Flag were analyzed by Western blot for STAT3 using anti-STAT3 or anti-Flag antibody. (**B**) TGF-β was measured by ELISA in conditioned medium of RPTECs treated ± collagen I (CI). Circles represent a single experiment performed in triplicate. Values are mean ± SD of at least 4 experiments and represent fold-changes versus vehicle-treated cells assigned as 1. Statistical analysis: 1-way ANOVA followed by Tukey’s multiple-comparison test. (**C**) TGF-β was measured by ELISA in conditioned medium of HK2 cells treated ± CI ± the STAT3 inhibitor S31-201 (10 μM). Values represent mean ± SD of at least 4 experiments and are expressed as fold-changes versus vehicle-treated cells assigned as 1. Statistical analysis: 1-way ANOVA followed by Dunnett’s multiple-comparison test versus CI-treated group.

To determine whether DDR1-mediated STAT3 activation contributes to the increased TGF-β production in collagen-treated cells, we generated RPTEC cells expressing a constitutively active form of STAT3 (STAT3C-Flag) (Figure 8A) and analyzed TGF-β production at baseline or after collagen treatment. At baseline, RPTECs expressing STAT3C secreted significantly more TGF-β than untransfected cells (Figure 8B). Treatment with collagen significantly increased TGF-β secretion in the control but not in RPTECs expressing STAT3C (Figure 8B). To further confirm the contribution of the DDR1/STAT3 axis in TGF-β production, we treated HK2 cells with collagen in the presence or absence of the STAT3 inhibitor S31-201. The inhibitor significantly decreased collagen-mediated TGF-β secretion (Figure 8C). Overall, these results suggest that collagen-induced activation of DDR1 results in tyrosine phosphorylation of STAT3, which in turn promotes TGF-β production.

## Discussion

**Figure 9:**
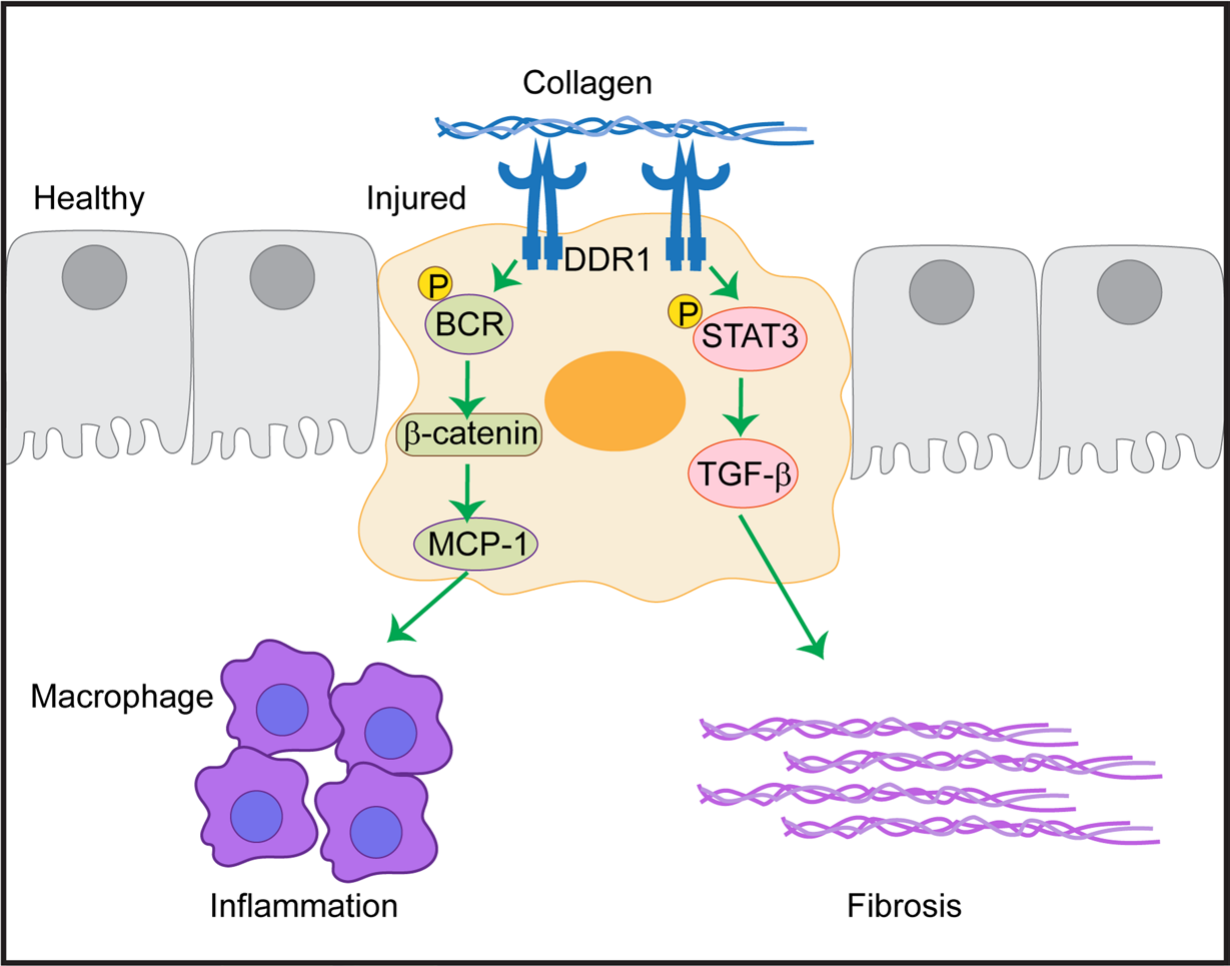
Schematic representation of DDR1-mediated proinflammatory and profibrotic signaling. Upregulation of DDR1 in injured RPTECs leads to a) phosphorylation of BCR, thus enabling β-catenin to regulate the production of proinflammatory MCP-1; and b) phosphorylation of STAT3 that induces the expression of the profibrotic TGF-β.

In the present study, we showed that DDR1 contributed to AKI and development of tubulointerstitial fibrosis by promoting both inflammatory and fibrotic signaling. These effects were mediated by increased DDR1 expression and activation in the kidney cortex, particularly in injured proximal tubules. We showed that DDR1 promoted BCR phosphorylation, thus removing its inhibitory effect on β-catenin and driving MCP-1 production by RPTECs. In addition, we showed that DDR1 promoted STAT3 activation, a key step in the regulation of profibrotic TGF-β. Thus, DDR1 contributed to inflammation and fibrosis by regulating production of MCP-1 and TGF-β by RPTECs (Figure 9).

Loss of DDR1 attenuates fibrosis in a mouse model of kidney injury induced by hypertension or partial renal ablation as well as in the Alport mouse model (9, 17, 26). Moreover, in kidney injury models associated with substantial inflammation, like UUO and nephrotoxic serum nephritis, loss of DDR1 reduces overall inflammation and fibrosis (6, 7). We showed that mice lacking DDR1 had reduced collagen deposition when fibrosis developed after severe AKI. Together, these data strongly support a profibrotic role of DDR1 in kidney injury. Furthermore, our observation of renal expression of both collagen I and activated DDR1 at d28 after injury suggests the presence of a vicious cycle where collagen I activates DDR1, which in turn promotes collagen production. Thus, our data support the hypothesis that DDR1 functions as an amplifier of the initial injury and thus exacerbates fibrotic responses, as previously suggested (10). A limitation of global *Ddr1-KO* mice is that it is difficult to determine whether the reduced fibrosis is due to overall reduced AKI or whether DDR1 affects both the acute and chronic injury separately. To answer this question, an inducible mouse model where DDR1 is selectively deleted in the proximal tubule after AKI is required. The *Ddr1*^tm1a^ mouse we obtained from European Conditional Mouse Mutagenesis (EUCOMM) represents an excellent tool to answer this key question. To this end, this mouse can be converted into a conditional ready *Ddr1*^tm1c^ floxed mouse upon breeding with a flippase recombinase transgenic mouse and then crossed with an inducible proximal tubule selective Cre mouse.

The contribution of DDR1 to acute injury and inflammation is not well understood. We provide evidence that DDR1 plays a role in our model of AKI. DDR1 expression is upregulated in kidney cortices at d3 after severe AKI, and *Ddr1-KO* mice have attenuated acute tubular injury, inflammatory cytokine production, and overall inflammation. These data are consistent with the finding that early (d4 after injury) antisense oligonucleotide-mediated DDR1 depletion is more efficacious than late (d8 after injury) DDR1 depletion in an NTS nephritis mouse model, where it decreases inflammatory cytokine production and inflammatory cell infiltration (8).

Despite the accumulating evidence that DDR1 promotes inflammation and fibrosis and, as such, is a promising therapeutic target, the mechanism whereby DDR1 exerts its biological function is poorly understood. This is in part because DDR1 signaling is cell type and context dependent. Our findings that DDR1 is upregulated in the renal cortices of mice subjected to severe AKI; it is expressed by proximal tubule cells in vitro (present paper and ref. 11); and it is upregulated in proximal tubules in patients with AKI who received a kidney transplant (11) as well as in mice subjected to AKI (present paper), clearly suggest that DDR1 expression on proximal tubule cells contributes to disease.

It is not clear whether DDR1 is also expressed on macrophages and directly promotes their migration to the site of injury. DDR1 was shown to be expressed on infiltrating macrophages in kidneys of mice subjected to UUO. By contrast, no DDR1 expression was found on the macrophages in angiotensin-induced kidney injury, and yet genetic deletion of DDR1 resulted in an overall reduction of inflammatory cell infiltration (7, 9). This result, together with the finding that DDR1 is not expressed on infiltrating inflammatory cells in patients with interstitial nephritis (4), suggests that DDR1 expressed on resident cells is responsible for the proinflammatory effects. We showed that DDR1 regulates MCP-1 production in RPTECs, which suggests that this is a major mechanism whereby macrophages are recruited to the site of injury.

Using BioID combined with proteomics, we found BCR to be the main target of DDR1 upon collagen-induced activation. BCR was identified as a DDR1 target in colon carcinoma cells using quantitative phosphoproteomics to identify proteins phosphorylated in response to DDR1 activation (18). Thus, BCR has been identified as a target for DDR1 in 2 independent studies using 2 different approaches. We showed that DDR1 phosphorylates BCR at Tyr177 in RPTECs, a site that is critical for regulating BCR interaction with binding partners like β-catenin (19). In cells stimulated with collagen, BCR phosphorylation was sustained, similar to DDR1 phosphorylation, and required DDR1 kinase activity, suggesting that BCR is downstream of collagen-induced DDR1 activation. Importantly, we showed that BCR was significantly phosphorylated in 3d injured kidneys in WT mice compared with *Ddr1-KO* mice, again suggesting that the DDR1/BCR axis contributes to injury.

The role of BCR in inflammation is controversial because BCR promotes and inhibits inflammation in a cell-dependent manner. To this end, BCR-deficient mice develop more severe septic shock after bacterial endotoxin challenge, and neutrophils isolated from these mice showed an increase in the respiratory burst (27). In contrast, in endothelial cells, BCR depletion suppresses the production of proinflammatory IL-6 (22). Our findings that BCR downregulation in RPTECs increased MCP-1 production, while BCR overexpression decreased MCP-1 production, indicate that BCR exerts an antiinflammatory role in the kidney.

In addition, we showed that unphosphorylated BCR modulated MCP-1 production by inhibiting β-catenin activity. Our data demonstrated that a) BCR depletion increased β-catenin nuclear translocation, consistent with BCR functioning as a negative regulator of β-catenin activation; b) increased MCP-1 production in BCR depleted cells was reversed by inhibition of β-catenin activation; and c) the increase in MCP-1 production after collagen-induced DDR1 activation/BCR phosphorylation was reversed by treatment with IWR1-endo. The WNT/β-catenin signaling has been implicated in several inflammatory diseases, and β-catenin activates the MCP-1 promoter (28). Consistent with our data, a recent study using a mouse expressing stabilized β-catenin in renal tubules, “Tubcat” mouse, showed that β-catenin activation in tubules promotes tubulointerstitial macrophage infiltration and MCP-1 production in a protein overload model of injury (29). Interestingly, β-catenin and MCP-1 colocalize in the tubules of the Tubcat mouse. This result, together with our finding that DDR1 promotes MCP-1 production in RPTECs and is upregulated in human injured proximal tubules (11), supports the idea that DDR1 expression in injured proximal tubules promotes inflammatory cell infiltration by enhancing the secretion of proinflammatory cytokines. Whether the DDR1/β-catenin axis plays a role beyond inflammation is not clear. The lack of fibrosis or activation of epithelial-mesenchymal transition markers in the Tubcat mouse suggests that the DDR1/β-catenin axis primarily regulates inflammatory responses.

A central question is how DDR1 leads to the development of fibrosis after AKI. In this regard, DDR1 activation in RPTECs promotes the secretion of the profibrotic cytokine TGF-β and induces tyrosine phosphorylation of the profibrotic transcription factor STAT3. *Ddr1-KO* mice have reduced renal levels of TGF-β in the UUO and NTS nephritis injury models (6, 7), but whether this reduction was due to attenuated injury or a direct role for DDR1 in promoting TGF-β secretion is unclear. Our finding that in RPTECs collagen induces TGF-β production, and this effect is inhibited by the DDR1 inhibitor Cmp-1, clearly suggests that DDR1 directly promotes TGF-β production. We also showed that activation of STAT3 is a key step in mediating DDR1-induced TGF-β secretion. Consistent with this finding, STAT3 activation increases TGF-β expression and promotes liver fibrosis in mice (30). In intestinal smooth muscle cells isolated from patients with Crohn’s disease, STAT3 activation increases TGF-β, connective tissue growth factor, and collagen I gene expression (31). Interestingly, in fibrotic skin of patients with systemic sclerosis, TGF-β downregulates the expression of SOCS3, which results in activation of STAT3. In turn, activated STAT3 promotes fibroblast-to-myofibroblast transition, collagen production, and fibrosis (32).

DDR1 has been shown to both activate and inhibit STAT3 in a cell type– and context-dependent manner. For instance, DDR1-mediated STAT3 activation is required for bladder tumor cell colonization to the lung (33) and for metastatic reactivation of breast cancer cells (34). In contrast, in Madin-Darby canine kidney cells, DDR1 inhibits Stat3 phosphorylation by activating the protein tyrosine phosphatase SHP2, thus inhibiting cell migration (35). In this study, we showed that a) STAT3 phosphorylation increased primarily in the proximal tubules of WT but not *Ddr1-KO* mice after AKI; and b) DDR1 activation increased STAT3 phosphorylation in RPTECs, and this event was reduced by treatment with the selective DDR1 inhibitor Cmp-1. Thus, we propose that after kidney injury, DDR1-mediated STAT3 activation may promote a vicious cycle whereby activated STAT3 promotes TGF-β production, which in turn further increases STAT3 activation, thus contributing to fibrosis.

In conclusion, DDR1 inhibition represents an attractive therapeutic option for kidney diseases. Successful inhibition of this pleotropic receptor would ameliorate kidney inflammation regulated by the BCR/MCP1 axis and kidney fibrosis regulated by the STAT3/TGF-β axis.

## Methods

### IR injury with delayed nephrectomy.

*Ddr1-KO* mice, received from the Samuel Lunenfeld Research Institute, Mount Sinai Hospital, Toronto, Canada, were backcrossed onto the 129Sv/Ev background for more than 10 generations. 129Sv/Ev WT and *Ddr1-KO* littermates generated from *Ddr1*het × *Ddr1*het mating were used for the in vivo experiments. DDR1 mice on the C57Bl6/129Sv mixed background with a KO-first tm1a allele (*Ddr1*tm1a), were purchased from EUCOMM. This constitutive KO-first allele has a *LacZ-*neomycin resistance cassette inserted between exons 5 and 6 of the DDR1 gene (see Supplemental Figure 1) to provide a reporter for *Ddr1* expression. *Ddr1*^tm1a/+^ × *Ddr1*^tm1a/+^ crossing were established in order to obtained *Ddr1*^tm1a/tm1a^ mice, which do not express DDR1 and express β-gal. Genotyping was performed according to European Mouse Mutant Archive (EMMA)/Infrafrontier protocol using the primers described in Supplemental Figure 1. Twelve-week-old male mice underwent left renal pedicle clamping for 31 minutes and nephrectomy of the right kidney after 8 days as previously described (13, 14). Mice were euthanized at different time points as described in the text.

### Clinical parameters and morphology analysis.

To determine BUN levels, plasma was collected at d–1, d9, and d28, and BUN was measured using the QuantiChrom Urea Assay kit (BioAssay Systems) following the manufacturer’s instructions.

Kidneys were fixed in 4% paraformaldehyde and embedded in paraffin. Paraffin tissue sections were stained with periodic acid–Schiff (PAS) for evaluation of tubular injury. For analysis, nonoverlapping fields in the kidney cortex PAS-stained sections were scored by a renal pathologist (400× original magnification) unaware of group assignment as follows: 0 = no injury; 1 = 1%–25% of area injured; 2 = 26%–50%; 3 = 51%–75%; 4 = 76%–100%. Acute tubular injury parameters included loss of brush border, vacuolization and/or blebbing, sloughing of tubular epithelial cells, tubular cast formation, tubular dilation, or naked tubular basement membranes. Chronic tubulointerstitial injury was defined as a matrix-rich expansion of the interstitium with infiltrating interstitial cells and chronic tubular injury (intratubular casts, atrophy of tubular cells with flattened/simplified tubular epithelial cells with thickened tubular basement membranes). A minimum of 27 nonoverlapping kidney cortex areas were analyzed for each kidney.

For fibrillar collagen deposition, paraffin sections were stained with Picrosirius red, and images of nonoverlapping kidney cortices (200× original magnification) were quantified using ImageJ (NIH) as described (https://imagej.nih.gov/ij/docs/examples/stained-sections/index.html). Fibrillar collagen was expressed as percentage area occupied by Picrosirius red–positive structures/microscopic field. A minimum of 5 nonoverlapping kidney cortex areas were analyzed for each kidney.

### IHC.

Paraffin kidney sections from uninjured and injured mice were stained with anti-F4/80 (1:200, Abcam, 6640) or anti–α-SMA (1:200, Sigma-Aldrich, A5228) antibody followed by HRP-conjugated secondary antibodies (1:300, Jackson ImmunoResearch, 712-035-153 or 715-035-150) and Sigma-Aldrich Fast DAB chromogenic tablets (Sigma-Aldrich). The number of F4/80-positive cells was counted in 400× nonoverlapping microscopic fields (20 images/kidney), and values were expressed as a percentage of F4/80-positive fields. Images of kidney cortices stained with anti–α-SMA antibody (200× original magnification) were quantified using ImageJ (7–8 images/kidney) as described above, and values were expressed as a percentage of α-SMA–positive areas.

For pSTAT3 staining, paraffin kidney sections were incubated with anti–pY705-STAT3 antibody (1:100, Cell Signaling Technology, 9145) followed by incubation with goat anti-rabbit Alexa Fluor 647 (1:500, Invitrogen, A21245) and fluorescein-conjugated lotus tetragonolobus lectin (LTL, 1:50, Vector Laboratories, FL-1321-2). Images (200× original magnification) were collected using the Zeiss LSM880 confocal microscope, and the number of pSTAT3-positive cells in LTL-positive proximal tubules was evaluated using ImageJ (5 images/kidney). Values were expressed as a percentage of positive p-STAT3/total number of proximal tubule cells.

β-Gal staining was performed as previously described (36). Briefly, euthanized mice underwent intracardiac perfusion with ice-cold 10% buffered formalin (Thermo Fisher Scientific, SF100-4), and then the kidneys were dissected and 2 mm sections were fixed in 10% formalin for 1 hour at room temperature, washed with PBS, incubated in 30% sucrose (MP Biomedicals, 821713) for 12 hours at 4°C, and then embedded on OCT compound (Thermo Fisher Scientific, 23-730-571). Kidney sections were incubated with β-gal stain solution (phosphate buffer pH 7.4 containing 2 mM MgCl_2_, 5 mM EGTA, 0.01% sodium deoxycholate, 0.02% Igepal, 5 mM potassium ferrocyanide, 5 mM potassium ferricyanide, and 1 mg/mL X-gal) for 12 hours at 37°C and then fixed with 10% formalin for 5 minutes at room temperature. After washing with PBS, sections were incubated with FITC-conjugated LTL (1:100, Vector Laboratories, FL-1321) for 2 hours at room temperature, washed with PBS, mounted with anti-fade mounting medium containing DAPI (Invitrogen, P36931), and then analyzed under an epifluorescence microscope.

### Plasmids.

For this study, we used human *DDR1b*, here referred to as DDR1. To generate DDR1-BioID, human *DDR1* cDNA was amplified using the primers 5′-GATGGAATTCGGAGCTATGGGACCAGAGG-3′ and 5′-GCTGGATCCCCACCGTGTTGAGTGCATC using pRK5-DDR1 as a template, as previously described (17). The PCR product was digested with EcoRI and BamHI and cloned between the same sites in MCS-BioID-HA (Addgene plasmid 74224), a gift from Kyle Roux (37). To generate DDR1-Flagb-TurboID, human *DDR1* cDNA was released from DDR1-BioID with NheI and BamHI and cloned in the same sites in pcDNA3.1(+). Flag-TurboID was amplified from Flag-TurboID plasmid (Addgene plasmid 124646, a gift from Feng-Qian Li and Ken-Ichi Takemura) using the primers: 5′-TGCAGGATCCACCATGGATTA-3′ and 5′-TCGAGCGGCCGCCTATAGTTCT-3′. The PCR product was digested with BamHI and NotI and cloned into pcDNA3.1-DDR1.

Murine *BCR-*Flag–tagged cDNA was obtained from Origene (MR222863). pRCStat3C was previously described (38). pIRES-DDR1 and pIRES-DDR1-K655A were previously described (17).

### Cell culture, transfection, and retroviral infections.

HEK293 cells expressing DDR1, generated as previously described (17), were cultured in DMEM (Gibco, 11995-065) supplemented with penicillin/streptomycin, 10% FBS, and puromycin 2.5 μg/mL (Sigma-Aldrich). DDR1-K655A, HEK-DDR1-BioID, HEK-DDR1-Flag-TurboID, and HEK-Flag-TurboID were generated as previously described (17). Briefly, HEK293 cells (ATCC, CRL-1573) were transfected with 2 μg of the corresponding DNA construct using Lipofectamine 2000 (Life Technologies) and then cultured in media containing 2.5 μg/mL puromycin or 2 mg/mL G418 (RPI). Drug-resistant clones were sorted by FACS for comparable levels of DDR1 expression using an antibody to the DDR1 extracellular domain (MilliporeSigma, MABT333) as previously described (17).

Conditionally immortalized mouse RPTECs, generated as described (39), were maintained at 33°C in DMEM/F12 media (Gibco, 11330-032) supplemented with 2.5% FBS, 50 ng/mL hydrocortisone (Sigma-Aldrich, H0135), 5 μg/mL insulin/transferrin/selenium (Sigma-Aldrich, I1884), 6.5 ng/mL triiodothyronine (Sigma-Aldrich, T5516), 92 μg/mL D-valine (Sigma-Aldrich, V1255), penicillin/streptomycin (Thermo Fisher Scientific, 15070063), and 10 U/mL IFN-γ (Sigma-Aldrich, I17001). For experiments, IFN-γ was removed and cells were maintained at 37°C for 72 hours to allow differentiation. To generate BCR-Flag or Stat3C-Flag cells, RPTECs were transfected with 2 μg of the corresponding cDNA constructs using Lipofectamine 2000 and then cultured in the presence of G418 (2 mg/mL) as previously described (38). After 4–6 weeks, G418-resistant clones were analyzed for BCR-Flag or Stat3C-Flag expression by Western blot analysis.

BCR-shRNA and Cnt-shRNA cells were generated by incubating RPTECs with lentiviral transduction particle carrying BCR-specific shRNA and expressing GFP (Sigma-Aldrich, SHCLNV TRCN0000374363) or nonmammalian shRNA control (Sigma-Aldrich, SHC002V) followed by treatment with puromycin (2.5 μg/mL). Two weeks later, BCR-shRNA RPTECs were sorted for GFP expression, and BCR downregulation was verified by Western blot analysis.

Human kidney proximal tubule cell line HK2 (HK-2, ATCC CRL-2190) was cultured in DMEM/F12 supplemented with 10% FBS and penicillin/streptomycin.

Primary RPTECs were isolated from 8–12-week-old WT and *Ddr1-KO* mice as shown in Supplemental Figure 4. Briefly, the kidney cortex was minced into small pieces, digested with collagenase (3 mg/mL, Worthington, S3N6800)/Dispase (1 mg/mL, Gibco, 17105-041)/DNAase (0.1 mg/mL, Sigma-Aldrich, 11284932001) in PBS, and passed through a 40 μm strainer (Thermo Fisher Scientific, 22363547). RPTECs labeled with biotinylated LTL (Vector Laboratories, B-1325-2) were separated on anti-biotin microbeads (Miltenyi Biotec, 130-090-485) and seeded on collagen I–coated dishes (30 μg/mL) and grown in DMEM/F12 media supplemented with 50 ng/mL hydrocortisone, 5 μg/mL insulin/transferrin/selenium, 6.5 ng/mL triiodothyronine, 92 μg/mL D-valine, murine EGF (20 ng/mL, Peprotech, 315-09) penicillin/streptomycin, and 0.5% BSA (Sigma-Aldrich, A3059). Cells were used between passages 2 and 4.

### Inflammatory cytokine array and kinase array.

For analysis of the inflammatory cytokines in RPTEC supernatants, we used the Mouse Inflammation Antibody Array membrane kit (Abcam, ab133999) following the manufacturer’s instructions. Briefly, supernatants from RPTECs treated with vehicle (20 mM acetic acid) or collagen I (50 μg/mL, Corning) for 24 hours were clarified by centrifugation and then incubated with the blocked membranes overnight at 4°C. The next day, membranes were washed and incubated sequentially with biotin-conjugated anti-cytokines, HRP-conjugated streptavidin, and chemiluminescent detection reagents. For the identification of kinases phosphorylated in response to DDR1 activation, we used Human Phospho Kinase Array (R&D Systems, ARY003B) according to the manufacturer’s instructions. Briefly, HEK-DDR1b treated with vehicle or collagen I (50 μg/mL) or HEK-vector cells treated with collagen for 2 hours were collected, lysed, and incubated with the blocked kinase array membranes overnight. The next day, membranes were washed and incubated with detection antibody cocktails followed by streptavidin-HRP and chemiluminescence detection reagents. Positive signals were quantified by densitometry analysis.

### Immunofluorescence.

To determine β-catenin localization, RPTECs were seeded in 8 multi-well chamber slides (2 × 10^4^/well) in complete medium. Twenty-four hours later, the cells were incubated in serum-free media with vehicle (20 mM acetic acid) in the presence or absence of 30 μM IWR1-endo (Tocris, 3532). After 24 hours, cells were fixed with 4% PFA and blocked with 3% BSA and 0.3% Triton X-100 in PBS. Cells were incubated with anti–β-catenin antibody (1:500, Vanderbilt Antibody and Protein Resource Core) at 4°C for 12 hours, and then incubated with Alexa Fluor 555–conjugated secondary antibody (1:400, Invitrogen, A-21422). Slides were mounted with anti-fade mounting medium containing DAPI (Vector Laboratories) and analyzed under an epifluorescence microscope. Images (400× original magnification) were recorded, and the numbers of nuclear β-catenin–positive cells and DAPI-positive cells per microscopic field (9 fields with a minimum of 400 cells) were evaluated. Values were expressed as a percentage of nuclear β-catenin–positive cells per microscopic field.

For zonula occludens-1 (ZO-1) and aquaporin 1 (AQP-1) staining, primary RPTECs grown on a polycarbonate culture insert (0.4 μm pore size, Costar, 3401) were fixed with 4% paraformaldehyde (Electron Microscopy Sciences, 15714), blocked and permeabilized with 3% BSA and 0.2% Triton X-100 in PBS, incubated with anti-ZO1 antibody (1:50, Invitrogen, 40-2200) followed by Alexa Fluor 488–conjugated secondary antibody (1:400, Invitrogen, A21206) or Alexa Fluor 647–conjugated anti–AQP-1 antibody (1:1000, Abcam, ab225225) for 12 hours at 4°C, then incubated with DAPI (1:200, Cell Signaling, 4083) in PBS for 10 minutes at room temperature. Slides were mounted with Prolong Gold Antifade reagent (Invitrogen, P36930), and images were acquired using a Zeiss LSM 880 confocal microscope with a 63×/1.40 Plan-Apochromat (Oil) objective.

### Western blot analysis.

Cells were lysed using lysis buffer (Cell Signaling Technology) supplemented with protease and phosphatase inhibitor (Sigma-Aldrich); kidney tissue was lysed in RIPA buffer (Sigma-Aldrich) supplemented with protease and phosphatase inhibitors and dissociated using a tissue homogenizer (Omni International). Both tissue and cell lysates were sonicated and centrifuged at 12,900*g* for 10 minutes to remove debris, and 20–100 μg for cells or 200–300 μg for tissue were separated by SDS-PAGE, transferred onto nitrocellulose, and immunoblotted as indicated.

Nonnuclear and nuclear fractions were isolated from kidney cortex or from cells as previously described (11). Equal amounts of proteins (10–20 μg for the nuclear fraction and 40–50 μg for the cytosol fractions) were separated by SDS-PAGE and transferred onto nitrocellulose and immunoblotted as indicated.

For detection of biotinylated protein, membranes were blocked with 5% BSA (Cell Signaling Technology), and then incubated with HRP-conjugated streptavidin (Jackson ImmunoResearch). For immunoblotting, membranes were incubated with the following primary antibodies from Cell Signaling Technology: DDR1 (catalog 5583), pY513-DDR1 (catalog 14531), pY792-DDR1 (catalog 11994), BCR (catalog 3902), pY177-BCR (catalog 3901), Stat3 (catalog 4904), pY705-Stat3 (catalog 9145), β-tubulin (catalog 15115), β-actin (catalog 4970), NF-κB-p65 (catalog 8242), pS536–NF-κB–p65 (catalog 3033), GAPDH (catalog 2118), N-cadherin (catalog 13116), and PARP (catalog 9532). In some experiments, cells were incubated with Flag (Sigma-Aldrich, F3165), collagen I (MD Bioproducts, SKU 203002), AQP-1 (Abcam, ab65837), and Claudin-2 (Invitrogen, 32-5600) antibodies overnight at 4°C. Membranes were then incubated with the appropriate HRP-conjugated (Jackson ImmunoResearch) or IRDye-conjugated secondary antibody (LICOR) and bands were detected using enhanced chemiluminescence (Perkin-Elmer, NEL 104001) or the Odyssey CLx imaging system, respectively. Bands were quantified using ImageJ or software provided with the Odyssey CLx.

### IP.

HEK-Flag-TurboID and HEK-DDR1-Flag-TurboID cells treated with vehicle or collagen I for 2 hours were lysed (Cell Signaling Technology lysis buffer), sonicated, clarified by centrifugation, and then equal amounts of cell lysates (200 μg) from HEK-turboID or HEK-DDR1-turboID were precleared by incubation with protein A for 1 hour at 4°C, and then incubated with Flag M2 affinity beads (Sigma-Aldrich) overnight at 4°C. The next day, Flag-beads were washed with 50 mM Tris pH 7.5 containing 150 mM NaCl and 1% Triton X-100, eluted in sample buffer (62.5 mM Tris pH 6.8, 2% SDS, 10% glycerol), and analyzed by Western blot for phosphorylated and total DDR1 and for total BCR.

### Mass spectrometry analysis.

Cells expressing DDR1(HEK-DDR1BioID) or empty vector (HEK-BioID) were serum-starved and treated with biotin (50 μM) followed by acetic acid (20 mM) or collagen I (50 μg/mL) for 24 hours. Cells were lysed and biotinylated proteins isolated using Neutravidin-Agarose beads (Thermo Fisher Scientific, 29200). Proteins were eluted by denaturation in sample buffer at 95°C for 5 minutes. Shotgun proteomic of eluted proteins was performed by first partially resolving protein mixtures at about 1.5 cm using a 10% Novex precast gel. The protein region was excised and subjected to in-gel tryptic digestion to recover peptides. Resulting peptides were analyzed by data-dependent liquid chromatography with tandem mass spectrometry. Briefly, peptides were autosampled onto a 200 mm by 0.1 mm (Jupiter 3 μm, 300A), self-packed analytical column coupled directly to an LTQ linear ion trap mass spectrometer (Thermo Fisher Scientific) using a nanoelectrospray source and resolved using an aqueous to organic gradient. Both the intact masses (MS) and fragmentation patterns (MS/MS) of the peptides were collected in a data-dependent manner utilizing dynamic exclusion to maximize depth of coverage. Using SEQUEST (40), resulting peptide MS/MS spectral data were compared with and scored against predicted tryptic peptides from a canonical human protein database (Uniprot) to which common contaminants and reversed versions of each protein were added. Peptide spectral matches (PSMs) were collated, filtered, and compared using Scaffold (Proteome Software). A Fisher’s exact test was performed within Scaffold to evaluate aggregate PSM differences between DDR1 and the control.

### Real-time PCR.

For RNA isolation, renal cortices were disrupted in Lysis Matrix Tubes (MP Biomedicals) containing TRIzol (Life Technologies, 15596018), and then further cleaned using RNeasy isolation kit (Qiagen). RNA was quantified using a nanodrop, and then equal amounts of RNA (1 μg) were used for cDNA synthesis using iScript cDNA Synthesis kit (Bio-Rad, 170-8890). Quantitative real-time PCR was performed using iO SYBR Green Supermix using the Bio-Rad CFX96 thermal cycler as previously described (11). The following primers were used: *Ccl2* (MCP-1) forward 5′-CCCAATGAGTAGGCTGGAGA, reverse 5′-TCTGGACCCATTCCTTCTTG, *Havcr1* (KIM-1) forward 5′-AAACCAGAGATTCCCACACG, reverse 5′- GTCGTGGGTCTTCCTGTAGC, and *Gapdh* forward 5′-TGGAGAAACCTGCCAAGTATGA, reverse 5′-GAAGAGTGGGAGTTGCTGTTGA.

### ELISA.

MCP-1 and TGF-β in 24-hour–conditioned medium from RPTECs or human kidney cells (HK2, ATCC, CRL-2190) were quantified using an MCP-1 ELISA kit (Abcam, ab100721) or a TGF-β1 ELISA kit (R&D Systems, DB100B or MB100B) following the manufacturer’s instructions. Briefly, cells were plated in 6-well plates (3 × 10^5^ to 4 × 10^5^ cells/well) in complete medium. Twenty-four hours later, cells were incubated with serum-free medium containing 20 mM acetic acid or collagen I (50 μg/mL in 20 mM acetic acid, Corning). In some experiments, cells were incubated with the DDR1 inhibitor Cmp-1 (3 μM) synthesized as previously described (16), or the WNT/β-catenin inhibitor IWR1-endo (30 μM, Tocris), or the STAT3 inhibitor S31-201 (10 μM, MilliporeSigma) for 30 minutes prior to 24 hours of treatment with acetic acid or collagen I.

### Statistics.

Data are expressed as mean ± SD. Statistical analysis was done using GraphPad Prism 9. For comparison of 2 groups, we used a 2-tailed *t* test. For comparison of multiple groups, we used 1-way ANOVA followed by post hoc analysis using Dunnett’s or Tukey’s multiple-comparison test. Repeated measurements on the same mouse data were analyzed by 2-way ANOVA followed by Sidak’s multiple-comparison test. *P* values less than or equal to 0.05 were considered significant. For the mass spectral data, Fisher’s exact test was performed with the Scaffold viewer (Proteome Software) with the Benjamini-Hochberg method used to estimate threshold *P* values for significance.

### Study approval.

The in vivo experiments were approved by Vanderbilt University’s IACUC and performed according to institutional animal care guidelines and conducted in facilities accredited by the Association for Assessment and Accreditation of Laboratory Animal Care.

## Author contributions

CMB designed research studies, conducted experiments, acquired data, analyzed data, and wrote the manuscript. XZ, FCA, MZ, GB, and FB conducted experiments and analyzed data. MDC, MY, EL, and LG provided reagents and experimental supervision. HY and ABF acquired data. WHM conducted experiments, acquired data, and analyzed data. RZ provided reagents and wrote the manuscript. AP designed research studies and wrote the manuscript.

## Supplementary Material

Supplemental data

## Version 1 12/23/2021

In-Press Preview

## Version 2 02/08/2022

Electronic publication

## References

[1] Bucaloiu ID (2012). Increased risk of death and de novo chronic kidney disease following reversible acute kidney injury. Kidney Int.

[2] Gewin L (2017). Progression of chronic kidney disease: too much cellular talk causes damage. Kidney Int.

[3] Moll S (2018). Selective pharmacological inhibition of DDR1 prevents experimentally-induced glomerulonephritis in prevention and therapeutic regime. J Transl Med.

[4] Moll S (2019). DDR1 role in fibrosis and its pharmacological targeting. Biochim Biophys Acta Mol Cell Res.

[5] Borza CM (2015). Cell receptor-basement membrane interactions in health and disease: a kidney-centric view. Curr Top Membr.

[6] Kerroch M (2012). Genetic inhibition of discoidin domain receptor 1 protects mice against crescentic glomerulonephritis. FASEB J.

[7] Guerrot D (2011). Discoidin domain receptor 1 is a major mediator of inflammation and fibrosis in obstructive nephropathy. Am J Pathol.

[8] Kerroch M (2016). Protective effects of genetic inhibition of discoidin domain receptor 1 in experimental renal disease. Sci Rep.

[9] Flamant M (2006). Discoidin domain receptor 1 null mice are protected against hypertension-induced renal disease. J Am Soc Nephrol.

[10] Prakoura N (2019). Novel targets for therapy of renal fibrosis. J Histochem Cytochem.

[11] Chiusa M (2019). The extracellular matrix receptor discoidin domain receptor 1 regulates collagen transcription by translocating to the nucleus. J Am Soc Nephrol.

[12] Coelho NM (2017). Discoidin domain receptor 1 mediates myosin-dependent collagen contraction. Cell Rep.

[13] Skrypnyk NI (2013). Ischemia-reperfusion model of acute kidney injury and post injury fibrosis in mice. J Vis Exp.

[14] Scarfe L (2019). Long-term outcomes in mouse models of ischemia-reperfusion-induced acute kidney injury. Am J Physiol Renal Physiol.

[15] Gewin LS (2018). Renal fibrosis: primacy of the proximal tubule. Matrix Biol.

[16] Jeffries DE (2020). Discovery of VU6015929: a selective discoidin domain receptor 1/2 (DDR1/2) inhibitor to explore the role of DDR1 in antifibrotic therapy. ACS Med Chem Lett.

[17] Borza CM (2017). Discoidin domain receptor 1 kinase activity is required for regulating collagen IV synthesis. Matrix Biol.

[18] Jeitany M (2018). Inhibition of DDR1-BCR signalling by nilotinib as a new therapeutic strategy for metastatic colorectal cancer. EMBO Mol Med.

[19] Ress A, Moelling K (2005). Bcr is a negative regulator of the Wnt signalling pathway. EMBO Rep.

[20] Peters KL, Smithgall TE (1999). Tyrosine phosphorylation enhances the SH2 domain-binding activity of Bcr and inhibits Bcr interaction with 14-3-3 proteins. Cell Signal.

[21] Ress A, Moelling K (2006). Bcr interferes with beta-catenin-Tcf1 interaction. FEBS Lett.

[22] Meng J (2016). Breakpoint cluster region-mediated inflammation is dependent on casein kinase II. J Immunol.

[23] Alexis JD (2009). Bcr kinase activation by angiotensin II inhibits peroxisome-proliferator-activated receptor gamma transcriptional activity in vascular smooth muscle cells. Circ Res.

[24] Lu J (2009). Structure-activity relationship studies of small-molecule inhibitors of Wnt response. Bioorg Med Chem Lett.

[25] Chen B (2009). Small molecule-mediated disruption of Wnt-dependent signaling in tissue regeneration and cancer. Nat Chem Biol.

[26] Gross O (2010). Loss of collagen-receptor DDR1 delays renal fibrosis in hereditary type IV collagen disease. Matrix Biol.

[27] Voncken JW (1995). Increased neutrophil respiratory burst in bcr-null mutants. Cell.

[28] Mestdagt M (2006). Transactivation of MCP-1/CCL2 by beta-catenin/TCF-4 in human breast cancer cells. Int J Cancer.

[29] Wong DWL (2018). Activated renal tubular Wnt/β-catenin signaling triggers renal inflammation during overload proteinuria. Kidney Int.

[30] Ogata H (2006). Loss of SOCS3 in the liver promotes fibrosis by enhancing STAT3-mediated TGF-beta1 production. Oncogene.

[31] Li C (2015). Noncanonical STAT3 activation regulates excess TGF-β1 and collagen I expression in muscle of stricturing Crohn’s disease. J Immunol.

[32] Dees C (2020). TGF-β-induced epigenetic deregulation of SOCS3 facilitates STAT3 signaling to promote fibrosis. J Clin Invest.

[33] Lee YC (2019). Collagen-rich airway smooth muscle cells are a metastatic niche for tumor colonization in the lung. Nat Commun.

[34] Gao H (2016). Multi-organ site metastatic reactivation mediated by non-canonical discoidin domain receptor 1 signaling. Cell.

[35] Wang CZ (2006). A discoidin domain receptor 1/SHP-2 signaling complex inhibits alpha2beta1-integrin-mediated signal transducers and activators of transcription 1/3 activation and cell migration. Mol Biol Cell.

[36] Chiba T (2016). Retinoic acid signaling coordinates macrophage-dependent injury and repair after AKI. J Am Soc Nephrol.

[37] Kim DI, Roux KJ (2016). Filling the void: proximity-based labeling of proteins in living cells. Trends Cell Biol.

[38] Borza CM (2012). Inhibition of integrin α2β1 ameliorates glomerular injury. J Am Soc Nephrol.

[39] Elias BC (2014). The integrin β1 subunit regulates paracellular permeability of kidney proximal tubule cells. J Biol Chem.

[40] Eng JK (1994). An approach to correlate tandem mass spectral data of peptides with amino acid sequences in a protein database. J Am Soc Mass Spectrom.

