# Supplementary material for: DDR1 contributes to kidney inflammation and fibrosis by promoting the phosphorylation of BCR and STAT3
Source: JCI Insight. 2022 Feb 8;7(3):e150887. doi: 10.1172/jci.insight.150887 (PMC8855801; doi:10.1172/jci.insight.150887)
Supplement: Supplemental data [file jciinsight-7-150887-s214.pdf]

SUPPLMENTARY INFORMATION

Borza CM et at

Supplementary Table 1

Partial list of mass spectral identifications of biotinylated proteins in HEK-BioID and HEK-DDR1BioID cells treated with vehicle or collagen I for 24 hours. Fisher's exact test was performed between vehicle- and collagen-treated HEK-DDR1BioID cells. *Indicates p-value threshold adjusted for multiple testing as estimated using Benjamini-Hochberg correction.

Gene	Protein Accession #	Fisher's Exact Test (p-value): *(p < 0.00283)	Total Peptide Spectral Matches (PSMs)		
			HEK-DDR1BioID	HEK-DDR1BioID	HEK-BioID
			Vehicle	Collagen	Collagen
AHNAK	Q09666	0.0001	44	18	0
BCR	P11274	0.0001	0	22	0
HSP90AB1	P08238	0.0046	17	44	1
DDR1	Q08345	0.0058	135	125	0
ACACA	Q13085	0.013	78	131	86
FLNA	P21333	0.033	69	62	0
MCCC1	Q96RQ3	0.034	11	27	10
CACYBP	Q9HB71	0.04	4	0	0
PCCA	P05165	0.047	28	52	27
HSP90AA1	P07900	0.14	8	17	0
EPS15L1	Q9UBC2	0.17	0	3	0
EPS15	P42566	0.17	0	3	0
H4-16	P62805	0.2	2	0	3
CHORDC1	Q9UHD1	0.2	2	0	0
ANXA2P2	A6NMY6	0.2	2	0	0
PC	P11498	0.23	51	73	16
DDX3X	O00571	0.23	2	6	0
HDLBP	Q00341	0.24	3	1	0
EPB41L3	Q9Y2J2	0.25	9	7	2
IRS4	O14654	0.26	18	17	0
HSPA1A	PODMV8	0.3	33	35	1
INPPL1	O15357	0.31	0	2	0
AIP	O00170	0.31	0	2	0
PICALM	Q13492	0.31	0	2	0
VPS51	Q9UID3	0.31	0	2	0
MAP1B	P46821	0.31	0	2	0
DSG2	Q14126	0.31	0	2	0
IF4G3	O43432	0.32	2	5	0
RUVBL2	Q9Y230	0.35	8	13	0
CTTN	Q14247	0.4	2	1	0
EPB41L2	O43491	0.4	3	2	0
AFDN	P55196	0.42	2	1	0

ERBIN	Q96RT1	0.42	2	1	0
DVL3	Q92997	0.43	8	8	0
DVL2	O14641	0.44	12	17	0
ESYT1	Q9BSJ8	0.45	2	4	0
H2A1B	P04908	0.45	1	0	2
DNAJB1	P25685	0.49	5	5	0
DVL1	O14640	0.49	3	5	0
APBB1	O00213	0.51	4	4	0
UBB	P0CG47	0.56	11	14	1
HSPA8	P11142	0.56	6	8	0
FKBP4	Q02790	0.58	1	2	0
KIFBP	Q96EK5	0.62	3	4	0

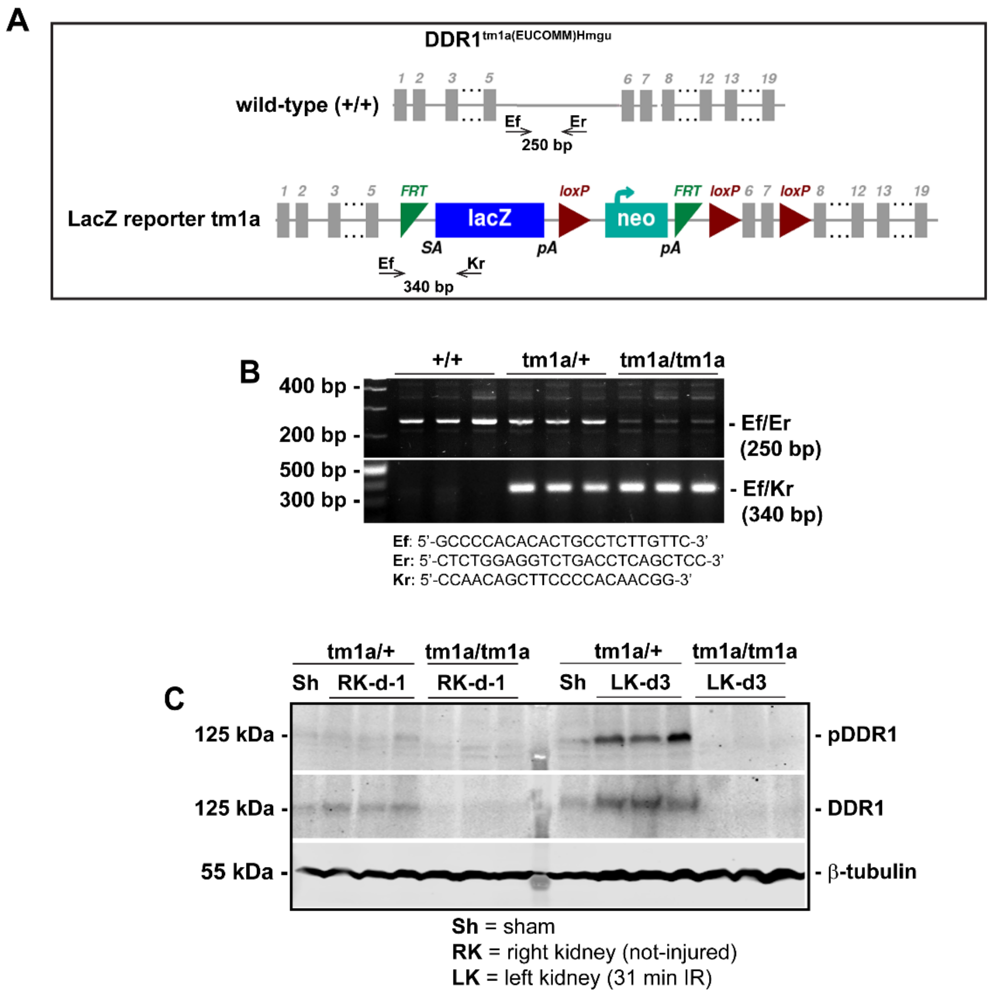

Supplementary Figure 1. Characterization of the DDR1^{tm1a} mouse

(A) Schematic diagram of the DDR1^{tm1a} knock-out first allele strategy. The cassette inserted between exons 5 and 6 contains flippase recombination enzyme (Flp)-recognition targets (FRT), a splice acceptor (SA), E. coli *LacZ* gene encoding the reporter enzyme β-gal, SV40 polyadenylation signal (pA), loxP sites and neomycin selection cassette. The third lox P site is inserted between exons 7 and 8. (B) PCR genotyping of the litter derived from *Ddr1*$^{+/tm1a}$ x *Ddr1*$^{+/tm1a}$ crossing. (C) pDDR1 and DDR1 levels were analyzed by Western blot in kidney cortices isolated from uninjured (d-1) or 3d injured mice.

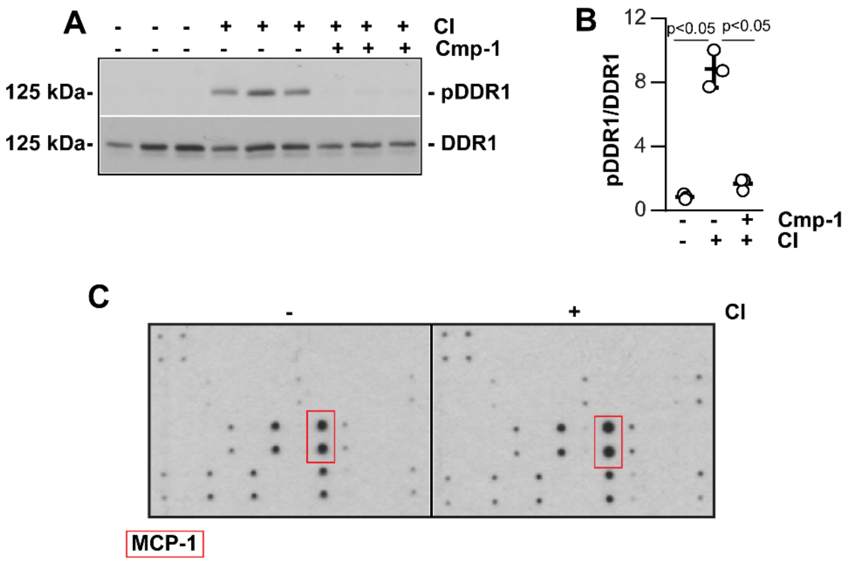

Supplementary Figure 2. DDR1 promotes MCP-1 production in RPTECs

(A) Western blot analysis of total and activated DDR1 in RPTECs treated with vehicle or collagen I (CI, 50 µg/ml) for 24 hours in presence or absence of the DDR1 inhibitor Cmp-1 (3 µM). This is a representative of two experiments each performed in triplicates. (B) pDDR1 and DDR1 bands were quantified by densitometry and pDDR1 is expressed as pDDR1/DDR1 ratio. Values show mean ± SD of one representative experiment performed in triplicates. Statistical analysis: One-way ANOVA followed by Dunnett's multiple comparison vs CI-treated cells. (C) Twenty-four hours conditioned medium from vehicle- or collagen I-treated RPTECs was analyzed for inflammatory cytokines using an inflammatory cytokine array (Abcam, ab133999). The signal corresponding to MCP-1 is shown in the red box. The black vertical line separates two membranes that were developed at the same time.

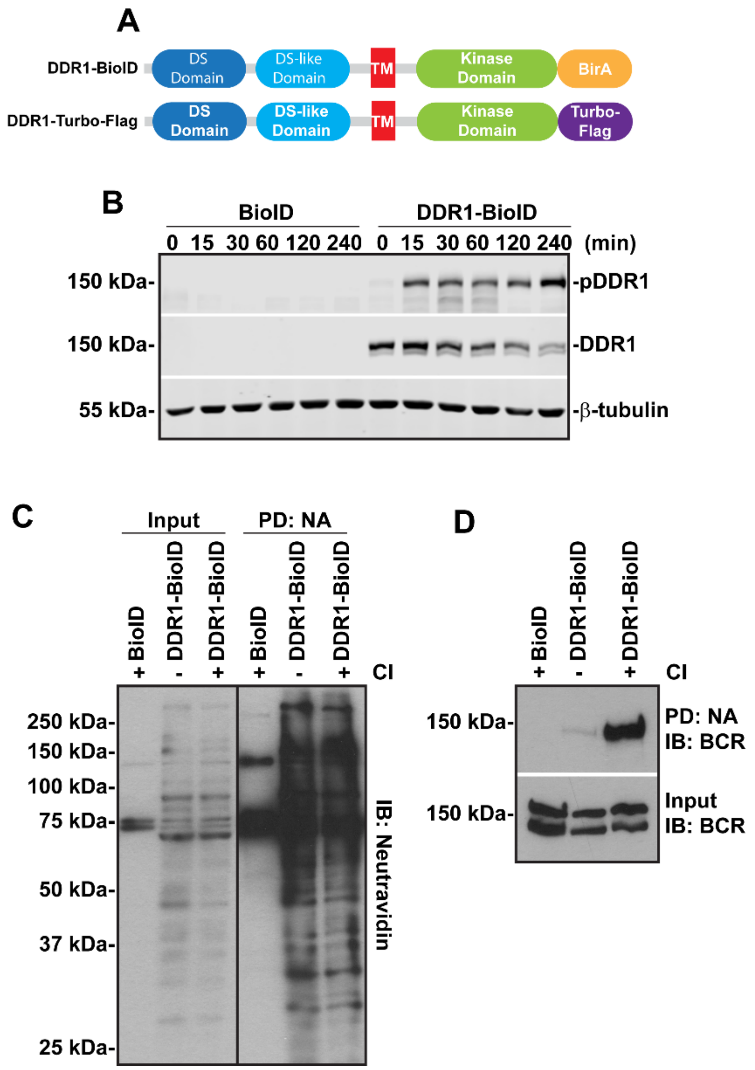

Supplementary Figure 3. Identification of BCR by BioID
(A) Overview of the DDR1-BioID and DDR1-Turbo-Flag constructs which contain the biotin ligase BirA or Flag-Turbo fused to the C-terminus of the *DDR1* cDNA. (B) HEK-DDR1-BioID cells were treated with collagen I (50 ug/ml) for the time indicated and then analyzed by Western blot for levels of total and phosphorylated DDR1. (C, D) HEK cells expressing BioID or DDR1-BioID were treated with vehicle or collagen I (50 μg/ml) for 24 hours and cell lysates were subjected to neutravidin (NA) pull-down (PD) followed by Western blot (IB) with HRP-streptavidin (C) or anti-BCR antibody (D). The black vertical line in C separates noncontiguous lanes run on the same gel.

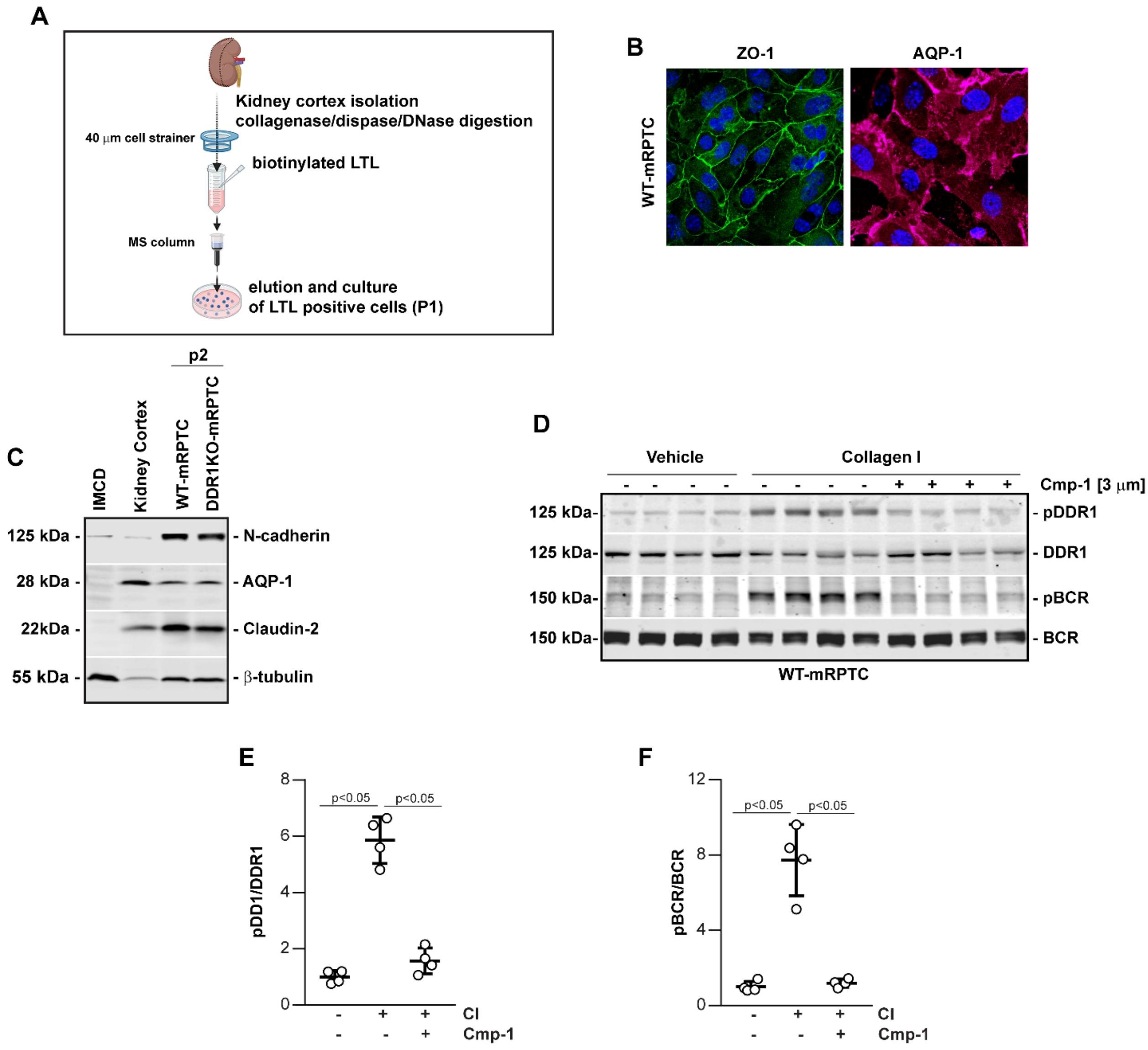

Supplementary Figure 4. Isolation and characterizations of primary murine renal proximal tubule cells

(A) Workflow of mouse primary renal proximal tubule cells (mRPTEC) isolation from mouse kidney. (B) mRPTECs isolated from WT mice were grown on transwells and then stained with anti-ZO-1 or anti-AQP-1 antibody, as described in the Methods. (C) mRPTECs isolated from WT and *Ddr1*KO mice were analyzed by Western blot for expression of proximal tubule markers AQP-1, Claudin-2 and N-cadherin. Lysates from mouse kidney cortex or inner medullary collecting duct cells (IMCD) are included as positive and negative control, respectively. (D) mRPTECs isolated from WT mice (n=4) were treated with vehicle or collagen I (50 μg/ml) in the presence or absence of the DDR1 inhibitor Cmp-1 (3 μM). After 2 hours, cell lysates were analyzed by Western blot for levels of phosphorylated and total, DDR1 and BCR. (E, F) pDDR1, DDR1, pBCR and BCR bands were quantified using the software provided by Odyssey CLx imaging system. Circles represent individual kidneys and the bars show mean ± SD. Statistical analysis: One-way ANOVA followed by Dunnett's multiple comparison test vs CI-treated cells.

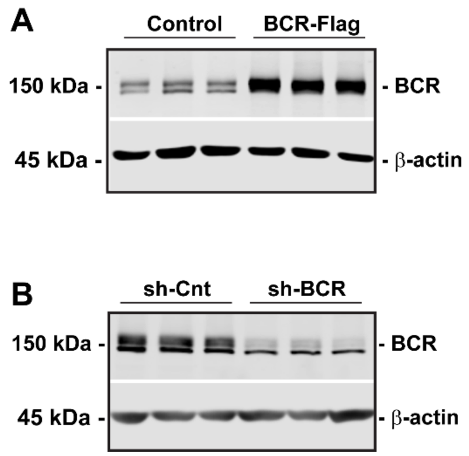

Supplementary Figure 5. Regulation of BCR levels in RPTECs
RPTECs untransfected (Control) or transfected with BCR-Flag (A) or infected with control (Sh-Cnt) or BCR (sh-BCR) shRNA (B) were analyzed by Western blot for levels of total BCR.

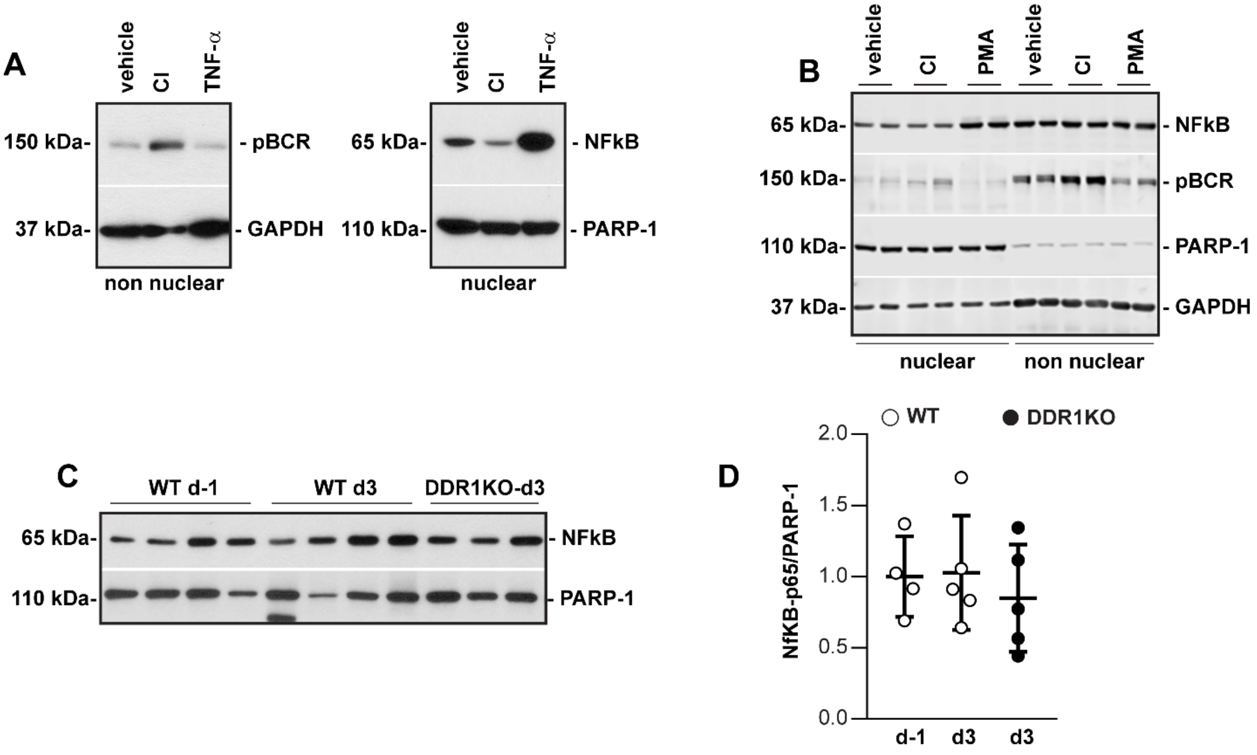

Supplementary Figure 6. DDR1 activation does not induce NFkB nuclear translocation
(A) HEK-DDR1 cells were treated with vehicle or collagen I (50 µg/ml) or TNFα (50 ng/ml, Sigma) for 10 minutes, (B) RPTECs were treated with vehicle, collagen I or phorbol myristate acetate (PMA, 100 ng/ml, Sigma) for 30 minutes. Nuclear and non-nuclear fractions were prepared as described in the Methods and analyzed by Western blot for levels of phosphorylated BCR (non nuclear) and NFkBp65 (nuclear). GAPDH and PARP-1 were used to verify loading and purity of the fractions. (C) Nuclear fractions from kidneys of WT and *Ddr1*KO mice uninjured, d-1 or 3 days after injury were analyzed by Western blot for levels of NFkBp65. (D) NFkBp65 and PARP-1 bands were quantified by densitometry and NFkBp65 levels are expressed as NFkBp65/PARP-1 ratio. Each circle represents and individual kidney d-1, WT n=4 d3 WT n=3, Ddr1KO n=5 and values are mean ± SD.

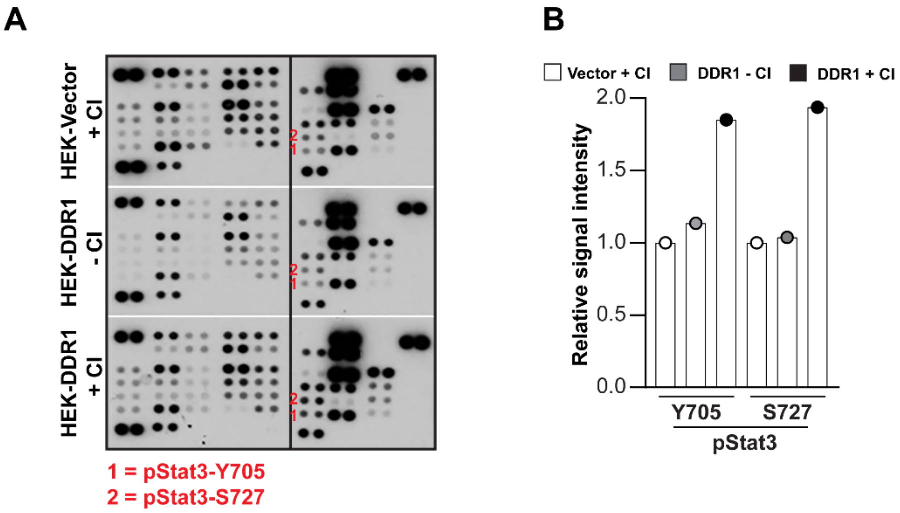

Supplementary Figure 7. Collagen-induced DDR1 activation promotes STAT3 activation
HEK-vector or HEK-DDR1 cells were treated with vehicle or collagen I (CI, 50μg/ml) for 2 hours and cell lysates (600 μg) were analyzed using a kinase array kit (R&D systems, ARY003B) according to the manufacturer's instructions. The black vertical line separates two membranes that were developed at the same time. (B). Average signal corresponding to phosphorylated Tyr705-STAT3 or Ser727-STAT3. Duplicate dots were quantified by densitometry analysis and expressed as a ratio to the reference spot. Data represent the fold change relative to HEK-vector + CI (assigned as one).
